# Immunological Mechanisms of Metal Allergies and the Nickel-Specific TCR-pMHC Interface

**DOI:** 10.3390/ijerph182010867

**Published:** 2021-10-15

**Authors:** Franziska Riedel, Marina Aparicio-Soto, Caterina Curato, Hermann-Josef Thierse, Katherina Siewert, Andreas Luch

**Affiliations:** 1 Department for Chemicals and Product Safety, Federal Institute for Risk Assessment, Max-Dohrn-Straße 8-10, 10589 Berlin, Germany; Marina.Aparicio-Soto@bfr.bund.de (M.A.-S.); Caterina.Curato@bfr.bund.de (C.C.); Hermann-Josef.Thierse@bfr.bund.de (H.-J.T.); Katherina.Siewert@bfr.bund.de (K.S.); Andreas.Luch@bfr.bund.de (A.L.); 2 Institute of Pharmacy, Freie Universität Berlin, Königin-Luise-Straße 2, 14195 Berlin, Germany

**Keywords:** immunotoxicology, metal allergens, allergic contact dermatitis, T cells, T cell receptor, T cell epitopes, cross-reactivity

## Abstract

Besides having physiological functions and general toxic effects, many metal ions can cause allergic reactions in humans. We here review the immune events involved in the mediation of metal allergies. We focus on nickel (Ni), cobalt (Co) and palladium (Pd), because these allergens are among the most prevalent sensitizers (Ni, Co) and immediate neighbors in the periodic table of the chemical elements. Co-sensitization between Ni and the other two metals is frequent while the knowledge on a possible immunological cross-reactivity using in vivo and in vitro approaches remains limited. At the center of an allergic reaction lies the capability of a metal allergen to form T cell epitopes that are recognized by specific T cell receptors (TCR). Technological advances such as activation-induced marker assays and TCR high-throughput sequencing recently provided new insights into the interaction of Ni^2+^ with the αβ TCR-peptide-major histocompatibility complex (pMHC) interface. Ni^2+^ functionally binds to the TCR gene segment TRAV9-2 or a histidine in the complementarity determining region 3 (CDR3), the main antigen binding region. Thus, we overview known, newly identified and hypothesized mechanisms of metal-specific T cell activation and discuss current knowledge on cross-reactivity.

## 1. Introduction

**Figure 1:**
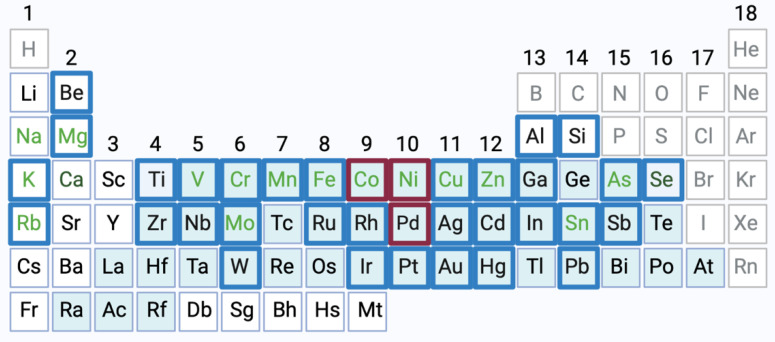
Metals implicated in allergic reactions. Metallic elements related to allergic reactions are highlighted in this periodic table of the elements (blue boxes, without lanthanides and actinides). Most of the metal allergens belong to the group of heavy metals (blue background) and some are (likely) essential trace elements (light green letters) [6,10]. Non-metallic elements are depicted in grey letters. This review focuses on general adverse and immunotoxic effects of nickel (Ni, Ni^2+^ ions), cobalt (Co, Co^2+^ ions) and palladium (Pd, Pd^2+^ ions) (highlighted in red) [6]. Created with BioRender.com.

Metals are present in many areas of industrialized life. People are exposed to metals through the environment, consumer products, workplaces and medical appliances such as implants or drugs. One of the most common immunotoxic effects of metals is their ability to act as allergens, often affecting the skin causing allergic contact dermatitis (ACD) [1,2,3]. ACD represents the characteristic example of a T cell-mediated delayed-type hypersensitivity response (type IV allergy). A multitude of metallic elements has been associated with allergic reactions, among them nickel (Ni, Ni^2+^ ions), cobalt (Co, Co^2+^ ions) and palladium (Pd, Pd^2+^ ions) (Figure 1) [4,5,6]. Metal allergens may interact with both the innate and adaptive immune system. Concerning the latter, the underlying mechanisms of allergic reactions include the formation of allergen-induced T cell epitopes recognized by specific T cell receptors (TCR) [7,8]. Since reactive chemicals are too small to be recognized by TCR or antibodies, binding to self-proteins by a process called “haptenization” is mandatory for the interaction with the adaptive immune system [9]. Thus, chemical allergies can be viewed as misguided adaptive immune responses to otherwise relatively harmless chemical exposures. Despite preventive regulations, metal allergies, especially Ni allergy, remain a significant public health burden [1,2].

In this review, we recapitulate the allergy-triggering mechanisms of Ni, Co and Pd identified from in vivo and in vitro studies. We focus on Ni because it is the most common contact allergen and there is abundant literature on Ni allergy. Further, we have selected the second most common metal allergen, Co, which is often used as a substitute for Ni in metal alloys. Besides, Ni-Co co-sensitization is observed in several epidemiological studies but currently there is little mechanistic evidence of a possible T cell cross-reactivity. In addition, we discuss the relatively rare allergen Pd. The current existing literature reports up to 80% Pd co-sensitization with Ni. Pd allergy is rarely diagnosed without additional Ni allergy and there is mechanistic evidence of cross-reactivity. In this context, we update the current understanding of metal-induced T cell epitopes and TCR cross-reactivity for Ni, Co and Pd.

## 2. Physicochemical Properties, Physiologic Functions and Toxicity of Ni, Co and Pd Ions

Metals are elements able to form cations via oxidation. The pure elements are solid under normal conditions (with the only exception being mercury) and possess high light reflectivity, hardness, ductility, malleability and an excellent heat and electric conductivity. The majority of the elements of the periodic table of the chemical elements (PSE) are metals (Figure 1, [11]).

Heavy metals are elements with at least a five times higher density than water and a high atomic weight [12]. This definition implies some controversial aspects and therefore some experts classify heavy metals as metallic elements with a density higher than 5 g/cm^3^ [13]. Popular belief associates the term heavy metal with some negative connotations linked to their potential toxicity. However, some heavy metals like Co are essential nutrients for humans whereas others, like Ni, are essentials for organisms such as bacteria and plants [14]. Heavy metals can play important roles in biochemical and physiological processes and their deficiency is associated with several diseases [15,16]. The toxic and carcinogenic molecular mechanisms of heavy metals are still not fully elucidated, but appear to be linked to a combination of common and unique features of every metal [12]. Some heavy metals can escape cell control mechanisms and bind cell constituents due to their chemical coordination and redox properties, thereby, for example, displacing original metals from their natural binding sites [17]. Other heavy metals lead to oxidative stress affecting several tissues, with liver and kidney being among the most critical ones [18]. Besides, the toxic properties of some heavy metals, leading to the formation of free radicals and reactive oxygen species (ROS), induce cell membrane and protein dysfunction linked to a malfunctioning immune system and potentially contribute to autoimmunity in humans [19].

According to the International Union of Pure and Applied Chemistry (IUPAC), a transition metal is an element with partially filled d-orbital (sub-shell) atoms or able to generate cations with an incomplete d-orbital. Metals from the first row of transition metals prefer tetrahedral to a square planar geometry (for example iron and Co). However, ions with incomplete d-orbitals (for example, Ni) prefer a planar conformation [20].

The number of ligands of a metal ion promotes a preferential spatial arrangement and influences its protein binding preferences [21]. The most thermodynamically favorable ligands for metal ions binding to proteins are the imidazole substituents in histidine, thiolate substituents in cysteine residues and carboxylate groups from aspartate or glutamate residues [22,23,24]. To date, global metal-binding proteins remain unknown while metalloproteomics is an emerging field of research [25].

Ni is a transition metal with the atomic (ordinal) number of 28 (molecular weight (MW): 58, 69) which belongs to group 10 of the PSE. It exists in different oxidation states (−1 to +4) but the most common is +2. Ni binds to proteins through the imidazole nitrogen of histidine and the thiol of cysteine [22]. The biological role of Ni in animals remains unclear, but some studies suggest its implication in reproductive and metabolic processes. Ni is a well-known essential co-factor of at least nine bacterial enzymes therefore indirectly influencing human health as part of the natural microbiome or pathogens [20,26,27,28]. Besides its high allergenic potential, Ni can also act as an immunotoxic and carcinogenic agent, can contribute to acute and chronic cardiovascular and respiratory diseases and it has revealed embryotoxic and teratogenic properties in animal studies [29,30,31,32,33,34].

Co is another transition metal with the atomic number of 27 (MW: 58, 93; group 9) and usually present at +2 oxidation state. Like Ni, it preferably binds to free histidine residues [35,36]. Co acts as a cofactor for several enzymes in humans and other organisms [37,38]. Exposure to Co may lead to metal allergy, chronic and acute respiratory diseases, metallosis and increased risk of cancer [35,39,40]. Metal implants made of Co alloys have been found to release high concentrations of Co^2+^ ions into the blood [41,42,43].

Ni and Co are commonly found together in nature and alloys. Co is placed next to Ni in the PSE (same period) with the same number of outer electrons and inner electron shells but a different number of lacking electrons of the inner shells, resulting in similar coordination properties. Although both metals show specific preferences regarding their amino acid binding, histidine and methionine are largely enriched in both metal binding peptides in bacteria, with a stronger histidine enrichment [44]. At certain metal concentrations, Co and Ni bind to the serum protein albumin, Co mainly at carboxylate and tyrosine residues and Ni at carboxylate groups. Co binding to albumin appears to be competitively inhibited by Ni, but Co did not inhibit Ni protein binding, indicating that Ni may have more binding sites [45].

Pd belongs to the same group of the PSE as Ni with an atomic number of 46 (MW: 106, 42; group 10). The most common oxidation state for Pd is +2 [46]. The catalytic properties of Pd are linked to the square-planar geometry of Pd^2+^ complexes [47]. Exposure to Pd via skin, oral cavity or respiratory tract can cause acute toxicity or hypersensitivity with respiratory and dermal symptoms [48,49,50]. Pd exhibited low genotoxicity in mammalian organisms and bacteria [51].

## 3. Metal Allergies

### 3.1. Exposure, Epidemiology and Regulatory Aspects

Exposure to metals can occur through different routes, such as dermal absorption, inhalation and ingestion. In daily life, the main exposure route by which ACD is triggered is the contact of metals with the skin via jewelry, clothes, consumer goods or the environment [2]. Besides, the surge of some metal nanomaterials in consumer products (mainly silicon, titanium, zinc and aluminum) also contribute to dermal and aerial exposures. The altered metal physiochemical properties and the unique immune effects of these nanomaterials may have important consequences in allergic processes [52]. The occupational exposure to metals, mainly via dermal or aerial routes, constitute an important health concern due to its involvement in ACD [2,53]. Metals can also encounter the human body systemically via implants and prosthetics. The current growing social demand for orthopedic and dental implants, joint arthroplasty and fixation devices may potentially contribute to the rise of contact and systemic allergies. Those devices are made of different alloys which can contain several metals including titanium, Ni, Co, chromium, iron, aluminum, vanadium and Pd among others [54,55]. The ingestion of metals via food and water is the dominant source of human metal exposure [56,57]. An allergic reaction can be triggered by dietary metal exposure in already sensitized individuals. In contrast, the significance for primary sensitization is still unclear [58,59]. The development of some pharmaceutical formulations with metals (mainly iron, zinc, vanadium, gold (Au), platinum and aluminum, among others) can also increase potential metal exposures and subsequent sensitization [15,60,61].

The number of patients with a metal allergy has recently been growing in general and surgical populations. Depending on the age and geographical location, approximately 20–27% of European adults are sensitized to at least one contact allergen as defined by positive patch test reactions and thus might be on the verge of developing ACD [1,62]. Most common are allergies to Ni (11.4%), fragrances (3.5%) and Co (2.7%) [1,6].

Allergen avoidance is the key to preventing sensitization, and ACD or other clinical symptoms. However, this may be difficult due to the ubiquity of the allergens, lack or shortcomings of identifying the substance in the environment, possible cross-reactions and low individual elicitation thresholds [52].

Since it is mainly dependent on exposure, ACD can affect all ages and may start at a very young age [63]. Some haptens (including Ni) have a prevalence peak in early adulthood and then show a decreased prevalence with aging [64]. The age-related variation may be attributable to different exposure conditions during the life span and a waning or reduced immune response with aging.

Although the use of allergens in consumer products, such as Ni in earrings, has been successfully regulated in recent years, the prevention of metal exposure needs further improvement given the severe socio-economic implications.

Ni is the most common cause of contact allergies in the general population worldwide (11.4% in Europe, 8.8–25.7% in China, 17.5% in North America) [1,65,66,67]. Ni is widely present in industrial and everyday items such as coins, cell phones, laptops and zippers as well as in some commonly consumed foods, air, soil and water [68]. Ni allergy is more prevalent in women (15.7–22.9% compared to 4.3–6.65% of men), probably due to their increased exposure to Ni through earrings and other jewelry [1,69,70]. Over time, Ni-induced ACD evolved from an occupational disease to a common form of ACD among both adults and children [71]. Several endogenous (e.g., genetics) and external factors are involved in the development of ACD to Ni, but the exact mechanisms that lead to human sensitization remain unclear. Major factors seem to be the accumulated Ni skin dose (μg/cm^2^) along with the type of exposure, skin status, skin area, bioavailability in the skin, duration of the contact, previous dermatitis or other skin diseases, sweat involvement and possible combined irritant effects by Ni itself or associated with other irritants [72,73]. Even very low but increased Ni levels in ambient air have been linked to increased sensitization rates [74]. Because of the high sensitization rate to Ni salts, Ni regulations were first implemented in Denmark and Sweden in 1990 and 1994 by the EU Ni Directive and later the Registration, Evaluation, Authorization and Restriction of Chemicals (REACH) legislation [75,76,77,78]. Since then, Ni is not allowed in items inserted into pierced parts of the body unless the Ni release is less than 0.2 μg/cm²/week or, for articles in direct and prolonged contact with the skin where Ni release is greater than 0.5 μg/cm²/week [79]. The European Commission also regulated the content of Ni in ambient air, establishing a target value of 20 ng/mg^3^ [80]. Although the prevalence of Ni allergy has decreased since the implementation of restrictions, Ni is nowadays still the most common cause of ACD in the general population. This can be partly attributed to the lack of restriction regarding the frequent contact of consumers with everyday products containing Ni [81].

Ni regulation promoted the use of other metals in consumer products, causing a rise in the incidence of ACD to other metals [82,83]. Co is the second most common metal allergen. Patients tested with the European baseline series between 2015 and 2018 showed a prevalence of positive tested individuals of 5.4%. Women were more frequently affected than men (6.1% vs. 3.9%), but in contrast to Ni, Co ACD does not display any age pattern [70]. Co is used in various dental alloys, paints and coloring components of porcelain and glass, often in combination with Ni [84]. Although Co allergy is relatively common, the causative exposure remains unknown in 80% of patients [1]. Co content has recently been regulated by EU legislation including a temporary generic concentration limit (GCL) of ≥0.1% [85]. Besides, a new restriction proposal from French and Swedish authorities aims to reduce the risk of skin sensitization to chemical substances in textile and leather articles. Thus, the proposed concentration limits for Ni and Co in textile and leather products are, respectively, 130 and 110 mg/kg and 70 and 60 mg/kg [86].

The general population is sensitized to Pd mainly through dental appliances. Additionally, Pd is present in jewelry and industrial catalysts in the car industry because of its resistance to corrosion [2]. Although the use of Pd is increasing, it is not included in any standard test baseline series [87]. Out of 910 ACD patients in the USA, 12.1% tested positive for Pd. Among these patients, mouth mucosal diseases were more common than skin diseases [88]. PdCl_2_ is the most frequent (3%) allergen of the “dental metal series” detected in dental technicians with occupational ACD [89]. The incidence of Pd allergies has frequently been explained through cross-reactivity since isolated allergy to Pd is rare [87]. Currently there is no EU legislation to limit the amount of Pd in consumer products.

### 3.2. Case Reports

Nowadays, there are many ways to encounter metal allergens, resulting in different clinical symptoms. We here highlight five interesting metal allergy cases from the recent literature.

Case 1—Systemic Ni allergy syndrome [90].

A 48-year-old woman presented with an inflamed esophagus. Ni allergy was diagnosed through patch testing. A Ni-free diet reversed symptoms while an oral Ni challenge induced adverse gastrointestinal effects. An oral desensitization therapy with slowly increasing amounts of Ni led to a complete reversal of symptoms. This case illustrates how metal-containing foods may cause systemic symptoms in metal allergic patients. It is an indication that oral desensitization may work similarly to hypo-sensitization for protein-related allergies, for example to pollen.

Case 2—Severe implant complications due to tiny amounts of Ni in suture anchors [91].

A 36-year-old woman developed severe local skin hematoma two months after an epicondylitis-related surgical procedure during which titanium suture anchors were inserted. Skin transplant and topical steroid treatment failed while Ni patch testing was strongly positive (+++). Although the dimethylglyoxime (DMG) test was negative, inductively coupled plasma mass spectrometry (ICP-MS) showed a release of Ni from the suture anchors. Removal of the anchors reversed symptoms, impressively showing how small amounts of metals can lead to delayed, severe complications in an implant setting.

Case 3—ACD to Ni from green tattoo ink [92].

A 40-year-old woman presented skin excoriations in the green area of a tattoo, that worsened during Ni patch testing (+++). Laser ablation ICP-MS revealed Ni agglomerates in the dermis, providing support for Ni-induced skin symptoms from tattoo inks. Tattoo inks can cause severe long-term problems if an allergic reaction occurs, which may be many years after the tattoo was placed. Metal allergens can be part of undeclared tattoo ink contaminations while the original inks are often not available at the time clinical symptoms emerge [93].

Case 4—Occupational ACD to Co due to contaminated machine oil [94].

A 24-year-old engineer on a container ship developed severe oozing dermatitis on his hands due to skin contact with machine oil. ICP-MS revealed the presence of a very low amount of Co (2.4 ppm) while other methods (spot test, X-ray fluorescence) failed to detect Co. Patch testing against Co was strongly positive (+++). This case illustrates that Co ACD elicitation thresholds can be extremely low in real life settings, especially on potentially damaged skin. The proof of causative metal allergen exposure can thus be very challenging.

Case 5—Pd-induced skin granulomas from earrings [95].

A 28-year-old woman showed epithelioid granulomas on both earlobes several months after piercing. Patch testing was only weakly positive to Pd (+) but after four weeks the patient developed similar granulomas at the site of the patch test. ICP-MS showed Pd agglomerations at all affected skin sites. Since more specific diagnostic tools are missing (e.g., reliable in vitro tests), no distinction between a foreign body and an allergic reaction is possible at present. Thus, this case illustrates current diagnostic limitations.

## 4. Immunological Mechanisms and Diagnostic Approaches

### 4.1. Pathomechanism of ACD

#### 4.1.1. Sensitization Phase

Similar to every allergic reaction, the pathomechanism of metal allergies can be separated into two temporally distinct phases, the sensitization phase and the elicitation phase, which each require the activation of innate and adaptive immunity [9,96,97]. One or several initial encounters of the skin with an allergen may lead to sensitization. The extent to which a chemical needs to penetrate the stratum corneum remains unclear, since chemicals may enter via skin appendages like hair follicles or sweat ducts and Langerhans cells may extend their dendrites through the tight junction barrier and thus contribute to chemical uptake [98,99,100].

Irritant effects of chemical allergens initiate an inflammatory milieu in the skin, leading to dendritic cell maturation and migration to the skin-draining lymph nodes [101,102,103]. In the lymph nodes, dendritic cells then present allergen-induced T cell epitopes to T cells. Activated, antigen-specific T cells clonally expand and differentiate into effector and memory T cells that distribute globally and may form antigen-specific tissue-resident memory T cells (T_RM_) [96,104,105]. The irritant power of chemicals seems to correlate with their ability to act as contact sensitizers [106], while the molecular pathways are not yet fully explored. Two main underlying mechanisms have been proposed by which sensitizing chemicals, including metals, modulate innate immune responses. Sensitizers can bind to pattern recognition receptors (PRR) and thereby functionally mimic pathogen-associated molecular patterns (PAMP). In addition, the release of damage-associated molecular patterns (DAMP) can be triggered, e.g., via ROS production or binding to other cellular constituents, leading to inflammasome activation and apoptosis induction [8,107,108,109,110,111,112]. Ni generates ROS and binds to proteins promiscuously and concentration-dependently, as shown for the formation of T cell epitopes [7,113,114,115]. Thus, a multitude of cellular effects is observed upon Ni exposure in, e.g., dendritic cells. This includes cholesterol depletion, hypoxic and apoptotic signaling, nuclear factor erythroid 2-related factor 2 (Nrf2) pathway activation and production of inflammatory cytokines like TNFα, IL-1b, IL-6, IL-8, IL-12 or IFNα and -β [116,117,118,119]. This leads to further downstream effects, e.g., IL-6 and IL-12 supporting naïve T cell priming.

One important pathway of Ni signaling is it’s functional interaction with human TLR4, which depends on the binding to two primate-restricted histidines (H456 and H458) [117]. Ni-sensitized human TLR4 transgenic mice react to intradermal NiCl_2_ injection in the ear pinna with ear swelling, illustrating the capacity of human TLR4 to contribute to Ni sensitization in vivo. Still, Ni-induced signals differ slightly from those observed with the natural TLR4 LPS ligand, e.g., IL-6 or Il-12p40 secretion is lower [116,117]. Human TLR4 is not expressed on freshly isolated keratinocytes or Langerhans cells, which most likely form the first site of contact in the epidermis with exogenous agents. Ni, Co and Pd have been shown to bind and signal via TLR4 [120].

Besides the intrinsic irritant capacity of sensitizers, heterologous immune stimulation may play a yet to be determined role in human sensitization to Ni and, in general, to metal allergens. The allergy to Ni is strongly associated with skin injury, e.g., piercing and very low exposure concentrations [74,121]. Likewise, the vaccination to cow pox relies on skin damage [122]. In addition, high metal ion concentrations during patch testing do not sensitize which also argues for a role of heterologous immune stimulation [123].

From a translational point of view, it remains difficult to prevent or interfere with the sensitization process by pharmacological intervention, leaving regulatory restrictions as possible public health measurement.

#### 4.1.2. Elicitation Phase and T Cell Effector Responses

Upon exposure of a sensitized individual to the original or a cross-reactive allergen, memory T cells initiate a faster and more aggressive secondary immune response, which results in a cutaneous inflammatory reaction clinically recognized as ACD (elicitation phase). ACD symptoms appear mainly on the skin (e.g., eczema and redness) but gut or lung symptoms as well as responses in joints or the oral mucosa are possible, e.g., in allergy-associated implant failure [9,124].

In a sensitized individual, dendritic cells, macrophages, Langerhans cells or keratinocytes may take up haptens and present hapten-induced epitopes in situ to effector T and T_RM_ cells developed during the sensitization phase. In order to exert their effector functions at the site of chemical exposure, allergen-specific memory T cells need to enter the tissue. T cell infiltration may be triggered by innate signals resulting from the irritant capacity of a chemical allergen that, as in the sensitization phase, involves a multitude of cellular and molecular players [96,125]. In addition, the activation of local antigen-specific T_RM,_ formed during the sensitization phase or prior elicitation reactions likely contributes. The potent effector functions and important role of T_RM_ have become a major topic in recent years since these cells are considered the main mediators of a plethora of skin diseases such as psoriasis, alopecia areata or mycosis fungoides and they cannot be therapeutically depleted at present [126]. Hence, prevention and regulatory strategies constitute an effective way for ACD management. In mice, CD4+ and CD8+ skin T_RM_ cells protect from infection and antigen-specific clonotypes distribute globally and accumulate at sites of antigen exposure [105,127]. Gaide et al. showed enrichment of individual skin-resident T cell clonotypes during the months following diphenylcyclopropenone (DPCP) exposure in humans [104]. Schmidt et al. illustrated a more rapid recruitment of CD8+ T cells at skin sites of previous ACD reactions which is indicative of local T_RM_ formation [128].

The chemical-induced innate response leads to the release of T cell-attracting chemokines. Kish et al. showed that murine CD8+ Trm produce IFNγ and Il-17A within the first 3 h of allergen exposure, which then triggers CXCL1, CXCL2/MIP-2 production by keratinocytes causing neutrophil infiltration, and, ultimately in this intricate series of events, secretion of T cell attracting chemokines, e.g., CXCL9/10, CCL17, CCL20 and CCL27 [129]. T cell infiltration only starts approximately 24 h after antigen exposure (thus the term “delayed-type hypersensitivity”) [96]. The extent of bystander T cell infiltration, i.e., the fraction of T cells that enter the skin but have an unrelated antigen-specificity, remains unknown, but it was shown that antigen-specific T cells proliferate locally, leading to a solidification of the allergic state and T_RM_ formation. In a study with three patients, Kapsenberg et al. found 7–15% of skin infiltrating CD4+ T cells, cloned by limiting dilution cultures, to be Ni-specific in Ni-ACD [130].

Once the inflammatory milieu is established, many cell types may serve as APC for memory T cell activation including keratinocytes that upregulate MHC II upon, e.g., IFNγ exposure [131]. This T cell response is the body’s attempt to remove the chemical allergen in a way similar to a pathogen.

Both CD4+ and CD8+ T cells as well as regulatory T cells seem to contribute to the allergic reaction in human metal allergy and murine contact hypersensitivity models [118,132,133,134,135]. Metal-specific regulatory T cells have been detected and shown to attenuate Ni-specific immune responses [136,137]. However, many aspects remain unknown, such as the relative contribution of CD4+ and CD8+ T cell subpopulations in humans for different chemical allergens. Here, more research is needed to advance alternative diagnostic and predictive testing. Future in vitro assays, for example, activation-induced marker assays, could track these populations in metal allergies [115,138].

During Ni-associated immune responses, a fraction of Ni-specific T cells expresses the skin-homing marker cutaneous lymphocyte-associated antigen (CLA), MHC II as activation marker (human leukocyte antigen (HLA)-DR) and Ki-67, which indicates an active cell cycle [115,139]. These findings illustrate that relevant metal-specific T cells can be identified by blood-based assays in vivo.

Metal-induced cytokine production by T cells has been studied quite extensively. Most metal-specific T cells produce INF-γ, which likely reflects the general high frequencies of T helper cells (T_H_) 1 in human blood [140,141]. Increases in rarer T cell sub-populations, e.g., T_H_2, T_H_17, T_H_9 or IL-10-secreting T cells, may be easier to detect given generally lower background and have been associated with metal allergies [142,143,144,145,146]. Whether there is a common polarization pattern among metal-specific T cells remains unclear. Among a limited number of donors, Ni-specific T cells from allergic donors comprised either a higher percentage of IL-4 or IL-17A producing cells, hence there was no general polarization pattern observed as for T_H_1/T_H_17A dominated CMV-specific immune response [115].

Given the involved steps as well as cellular and molecular effectors during an elicitation response, several therapeutic options are conceivable, but most have not yet been transferred into practice. Prevention of exposure is the top priority measurement if possible and it may be supported using barrier creams. Once ACD has developed, the first line treatment is the topical use of corticosteroids that dampen the inflammatory immune response. Similar treatments, such as cyclosporine, azathioprine, methotrexate, psoralen or UVA, may also control inflammation and are used as second line approaches upon corticosteroids resistance. So far, biologics (e.g., a-IL17 therapy, [147,148]) or oral tolerance induction have not been included in guideline-based treatment [149,150]. 

### 4.2. Diagnosis of Metal Allergies

#### 4.2.1. Patch Testing as the Current Diagnostic Standard

So far, no causative therapy exists for metal allergies. Therefore, a precise diagnosis is crucial to avoid the chemical trigger and to prevent clinical manifestations of the disease. The in vivo method of patch testing was first applied in 1895 and is still the diagnostic standard today [149,151]. Patch testing aims to mirror the elicitation phase of ACD. Due to the high frequencies of positive patch test reactions, both Ni and Co salts are components of the European baseline series of contact allergens, which is used to diagnose contact allergy as a cause of clinically visible skin dermatitis in patients [70,151,152]. Occlusive applications of patch test allergens are deposited on the upper back for 48 h and diagnostic inspection follows after 48 h and 72 h with a standardized scoring of − and + to +++. Late readings, e.g., after 5–7 days, may yield additional positive reactions, especially in the case of metal allergies [153,154,155]. Patch test reactivity to Ni may decline if tested on other body areas than the upper back, e.g., on palms of the hands, which anatomically possess a thicker epidermis and less antigen-presenting cells (APC) [156].

Patch testing material contains Ni (II) sulfate hexahydrate (NiSO_4_ × 6H_2_O; 5% concentration (w/w)) and petrolatum (pet.) as a vehicle. For historical reasons, water-soluble Ni sulfate is employed which seems to be less irritant than Ni chloride [157,158].

For Co patch testing, Co (II) chloride hexahydrate (CoCl_2_ × 6H_2_O; 1% concentration (w/w)) pet. is used (0.4 mg/cm^2^). Pd allergy is often diagnosed with 1% Pd (II) chloride (PdCl_2_ pet.), e.g., in the standard testing series for dental metals in Germany. Interestingly, sodium tetrachloropalladate (Na_2_PdCl_4_) may represent a better patch test allergen as it reveals more positive reactions, likely due to enhanced skin penetration [159,160]. This illustrates how the choice of the allergen preparation, including “the stability and purity of the allergen, its physical form, and the homogeneity of its distribution throughout the vehicle” [161] and as well its release from the vehicle, e.g., from petrolatum, influences patch test results.

Patch testing reproducibility is somewhat limited, especially for weakly positive reactions and for metal allergens. For instance, the reproducibility of CoCl_2_ patch testing is only 35% [162,163,164,165,166,167,168]. In the case of patients with a clear history of metal allergy, a skin patch test is recommended before device implantation [169]. Nevertheless, there is no clear consensus regarding when and how to screen metal hypersensitivity before implant or prosthetic interventions. Besides, the correlation between patch test results and the prediction of implant failures is still controversial [55,170]. This could be due to a varying distribution of metal allergen-specific T_RM_ between the skin and target tissue of the implant.

In the case of concomitant diseases, e.g., “angry back” or “excited skin” syndrome, patch testing may not be possible. As another disadvantage of patch testing, the triggered local inflammation due to a positive patch test may enhance subsequent elicitation reactions in previously sensitized individuals as reported for human ACD [128,171].

In summary, patch testing has been proven very useful for over a century, but it is not a perfect tool. Therefore, diagnostic improvements, i.e., the development of reliable additional in vitro tests, are urgently needed.

#### 4.2.2. Challenges Associated with Diagnostic In Vitro Tests

**Figure 2:**
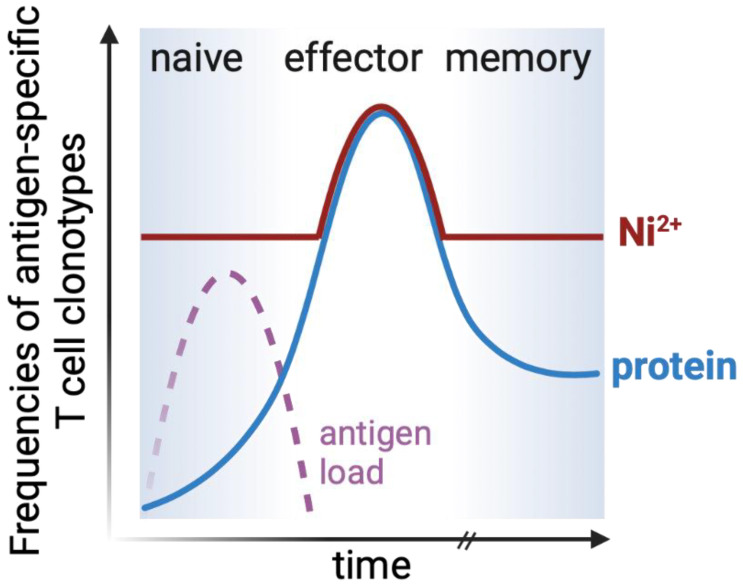
High frequencies of metal-specific T cells in blood interfere with diagnostic blood-based in vitro testing. Following adaptive immune responses, blood frequencies of protein-specific memory T cells are usually higher compared to those in the naïve T cell pool, even decades after antigen exposure (blue line). However, Ni-specific T cells are abundant in non-allergic individuals (red line), due to interactions with certain elements of the TCR repertoire (see Section 5.2) [115]. Therefore, only strongly increased frequencies of Ni-specific T cells may currently be associated with the allergic state. Adapted from [174]. Created with BioRender.com.

In vitro tests could overcome some of the above-mentioned limitations of patch testing. The aim of in vitro tests is to detect increased frequencies of antigen-specific memory T cells in the blood of allergic individuals similar to assays performed after vaccination to smallpox or yellow fever [172,173]. Yet, in vitro tests have not been officially implemented as diagnostic tools in ACD [149]. The difficulties of in vitro T cell tests for metal allergies seem to be mainly linked to the presence of rather high frequencies of metal-specific T cells in non-allergic individuals. The reactivity in non-allergic donors impedes the distinction of allergy-associated immune responses (Figure 2). In addition, T cell-based assays are labor and cost-intensive.

Ni is the most intensively studied metal allergen in vitro. Proliferation-based approaches, e.g., lymphocyte transformation or proliferation tests (LTT or LPT), Memory Lymphocyte Immunostimulation Assay (MELISA), or cytokine-secretion assays (e.g., Enzyme-linked-immuno-Spot Assay (ELISpot)) have been employed [6,175]. Our group recently showed that CD154 upregulation identifies the Ni-specific CD4+ naïve and memory T cell pool in a fast, comprehensive and quantitative manner [115]. Thus, the benefits of activation-induced marker assays may also be exploited in the field of chemical allergens, opening new avenues for the investigation of involved T cell responses [138,176].

Regarding Ni-specific T cells, often, similar responses have been observed in non-allergic and allergic individuals, especially for CD4+ T cells [89,115,132,177,178,179]. The frequency of Ni-specific CD4+ T cells correlates with the used Ni concentrations and varies between ~0.00005%, 0.02% and 0.1% at Ni^2+^ concentrations close to zero, ~40 µM, and 200 µM, respectively [115,132,180]. However, no concentration has been determined which optimally distinguishes allergic from non-allergic individuals [89,177,181]. Local in vivo concentrations in the skin or lymph nodes remain unknown. The use of high but non-toxic Ni^2+^ concentrations in vitro may completely capture the specific T cell pool while unrelated immune responses, e.g., to cytomegalovirus (CMV), do not seem to interfere [115].

Co seems to activate a similar or slightly smaller percentage of T cells compared to Ni, as judged from proliferation-based assays, [181,182,183] while Pd-specific T cell frequencies have hardly been investigated [89,184]. Unless limited dilution cultures are used, proliferation-based assays do not allow a direct assessment of the initial frequencies of reactive T cells. Cristaudo et al. detected IFN-γ-release by ELISpot after incubation of peripheral blood mononuclear cells (PBMC) with 14 µM PdCl_2_ and observed strongly increased responses in six allergic donors with ongoing oral symptoms and positive patch test reactions compared to 10 non-allergic controls [185]. This finding indicates that Pd-specific T cells can be detected similar to Ni- and Co-specific T cells in vitro. Of note, cytokine release assays capture only cytokine-secreting T cell subpopulations, not the complete antigen-reactive pool.

In some publications, high frequencies of metal-reactive T cells have been attributed to “unspecific mitogenic effects” [186,187]. Still, the specific restimulation of individual T cell clones and prevention of activation with major histocompatibility complex (MHC) blocking antibodies argues for a mainly TCR-mediated activation [115,188,189].

In summary, only some allergic individuals top background frequencies of metal-specific effector or memory T cells in blood-based in vitro tests (Figure 2). Further research is required to identify more distinctive, allergy-associated T cell subpopulations or, if tissue samples are available, in situ allergy-associated immune responses [175,190].

Future in vitro blood-based allergy tests should optimally detect outgrowth of different cytokine-producing T cell subsets and, if possible, additional markers of T cell activation. For this purpose, activation-induced marker assays are especially promising since they more likely report ex vivo phenotypes of allergen-specific T cells if combined with multiparameter flow cytometry compared to proliferation-based methods [115,176].

#### 4.2.3. Predictive Tests for Sensitizing Properties

Predictive tests aim to determine the sensitization hazard and, optimally, the potency, i.e., the dose (µg/cm^2^) required for sensitization. In mice and guinea pigs, Ni sensitization requires relatively high percutaneous doses or intradermal injection in the presence of costimulatory signals [118,191,192,193]. One underlying mechanism may be the lack of the primate-restricted histidine duplet (H456/H458) in murine and other rodent TLR4 [117]. Ethically questionable human experiments from the 1960s showed that exposure to NiCl_2_ sensitized 48% of individuals [194,195]. In real life, Ni allergy has been associated with very low exposure concentrations and skin injury (piercings) [74,196]. This indicates a role for heterologous immune stimulation and illustrates bottlenecks in current regulatory testing. Established in vitro tools do not assess heterologous immune stimulation, T cell activation, or experimentally validated cross-reactivity [197]. Future T cell-based assays may help to assess T cell activation for either allergen mixtures, metal alloys or both. These tests could possibly be based on activation-induced marker assays which offer many advantages compared to proliferation-based assays [115,138,176].

## 5. TCR Antigen Recognition, Metal-Induced T Cell Epitopes and Cross-Reactivity

### 5.1. General Considerations Regarding Antigen Recognition by TCR

Matzinger and Bevan were the first to postulate that a TCR can be activated by a variety of peptides presented by one MHC [198]. In the past, clonal selection theory suggested that each T cell selectively recognizes only one pMHC complex [199,200]. Cross-reactivity, also known as immune polyspecificity, is defined as the ability of a TCR to bind and functionally respond to more than one pMHC complex. Mason further extended the cross-reactivity conceptual framework stating that the TCR repertoire must be able to recognize all foreign peptides presented by MHC proteins [201]. MHC I molecules, which present antigens to CD8+ T cells, may theoretically present more than 12 × 10^11^ different peptides with a length of 10 amino acids (considering the presence of anchoring residues). MHC II, which present antigens to CD4+ T cells, may present even more peptides because these antigen-presenting molecules have an open-ended binding groove that can accommodate longer peptides [202].

These numbers of possible antigen structures vastly exceed the huge, but still limited numbers of different αβ TCR (≥10^8^, among ~10^12^ T cells) which result from V-(D)-J-recombination and are expressed in each individual [203,204]. Therefore, T cells may only provide complete immune coverage if each TCR detects many (foreign) peptides. Wooldridge et al. showed that a single autoimmune CD8+ T cell clone recognizes more than 10^6^ different artificial peptides with a length of ten amino acids on a single MHC I molecule [205]. Once the first multimolecular X-ray diffraction structures of complete TCR-pMHC complexes became available, details on TCR binding and non-mutually exclusive mechanisms underlying cross-reactivity have been discovered [206]. Currently, the STCRDab database lists 68 human TCR-pMHC I and 20 TCR-pMHC II structures (related structures from the same TCR were excluded; http://opig.stats.ox.ac.uk/webapps/stcrdab/, accessed on 13 October 2021 [207]). This still limited but steadily growing database only comprises one metal-specific TCR (beryllium (Be)-specific [208]) and one Ni-specific TCR (ANi2.3), for which a Ni-independent mimotope has been isolated [209]).

#### 5.1.1. TCR-pMHC Contact Points and General Binding Orientation

**Figure 3:**
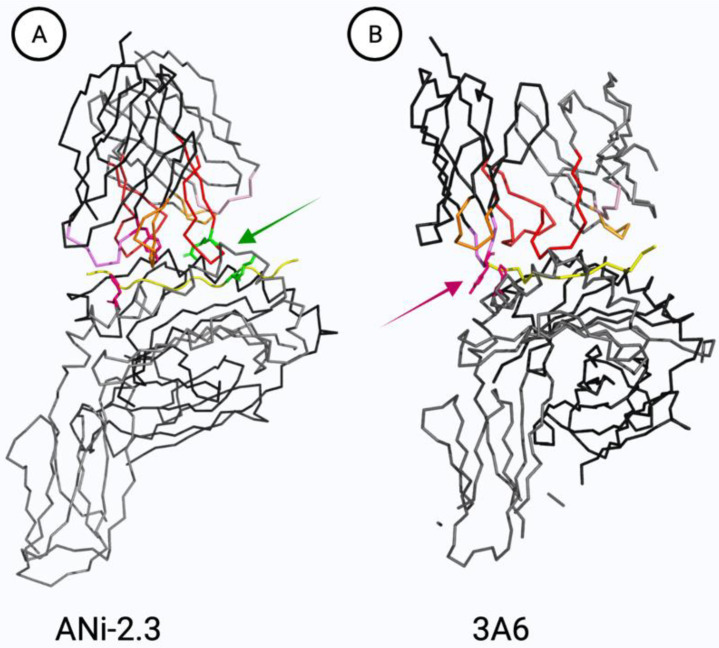
Structure of TCR-pMHC II complexes. TCR α- and β-chains (top) with CDR3 regions (CDR1, pink; CDR2, orange; CDR3, red) are positioned diagonally over MHC II α- and β-chains (bottom) and the presented peptide antigens (yellow). (**A**) Ni-specific TCR ANi-2.3 (TRAV8-TRBV19) in complex with a peptide mimotope (pdb ID code 4h1l). Lys_7_ likely replaces Ni in this structure with Asp_95_ being an essential contact point (green stick representation, [209]). (**B**) TCR 3 A6 (MBP 89-101-specific, TRAV9-2-TRBV5) represents the only available TCR-pMHC II structure of a human TRAV9-2 TCR (pdb ID code 1 zgl). His_81_ is a major TCR contact point in the MHC II β-chain and is in close proximity with Tyr_36_ in the TCR α-chain CDR1 of TRAV9-2 (pink stick representation). A recent study found that approximately 35% of Ni-specific TCR expressed TRAV9-2 compared to ~5% in the random repertoire [115]. The depicted TCR do not express a histidine in their CDR3 regions, which constitutes an independent recently discovered major Ni binding mechanism [115] (see Section 3.2). Created with Pymol.

In the typical αβ TCR binding mode, the TCR is oriented diagonally above the pMHC complex (Figure 3) [210]. The Vα domain lies over the amino-terminal end of the peptide (α2 helix MHC I, β1 helix MHC II) and the Vβ domain lies over the carboxy-terminal end of the peptide (α1 MHC helices). The contact points of the complementarity-determining regions (CDR) 1 and 2 to the MHC are mostly conserved [211]. The CDR3 regions of both, TCR α- and β-chain, are the most variable binding sites and are mainly in contact with the antigen peptide [210]. Details in the binding of the CDR loops to the peptide and the MHC may vary greatly for different TCR.

#### 5.1.2. Cross-Reactivity due to Conserved Binding Sites

The interaction between TCR and pMHC is only partly conserved and allows flexibility mainly within the borders of some general orientation and binding rules [202]. Several crystal structures from the same TCR binding to different pMHC complexes formed the concept of conserved interaction. This concept indicates that a small, conserved amino acid motif stabilizes the TCR interaction with the pMHC complexes. Outside of this motif, a large number of changes can be tolerated, allowing a TCR to identify many different binding partners [212,213,214]. Within the motifs, only a few changes in the amino acids are tolerated. The clonal dependence upon these interactions can vary [215]. Peptide binding to MHC is mainly independent of the TCR engagement and involves different interactions of anchoring amino acids with the MHC binding groove. Thus, it is possible to isolate peptide mimotopes from artificial randomized peptide libraries that may be enriched in MHC-anchoring amino acids at certain positions. Mimotope alignment then reveals the conserved amino acid motif for a given TCR and, by protein database mining, can elucidate the original unknown antigen because biological peptide variability is limited [209,213,216,217]. However, in metal allergies, the coordinative bond of the metal ion poses additional challenges in epitope identification [209].

#### 5.1.3. Cross-Reactivity due to Induced Fit

Another, complementary, concept for cross-reactivity is induced fit [218], also referred to as structural plasticity or conformational melding. During induced fit, the CDR loops and the pMHC complex cooperatively adjust and may undergo large conformational changes mainly without altering the overall docking orientation [219,220]. In general, the CDR3 loops change their conformation more than the other loops, the peptide or MHC [206,221,222,223,224].

An example for cross-reactivity is molecular mimicry. Pathogenic peptides may mimic self-peptides and thus evade immune recognition [225]. In molecular mimicry, ligands share structural and chemical features [226]. The cross-reactivity may be focused on so-called “hot-spot mimicry” where short amino acid stretches are identical between pathogen and self-peptide, showing the connection to the concept of conserved binding sides [227].

Cross-reactive T cells may have advantages and disadvantages. For instance, cross-reactive T cells may recognize antigen peptides from related pathogens and thus provide protection to heterologous infections (this seems limited in the current SARS-CoV-2 pandemic with respect to former common cold coronavirus (SARS-CoV-1) infection [228,229]). On the downside, cross-reactive TCR may contribute to autoimmunity [225].

### 5.2. Metal-Induced T Cell Epitopes

**Figure 4:**
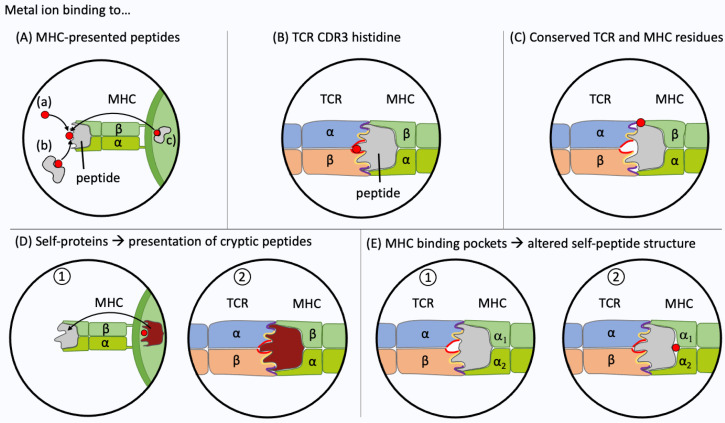
Metal recognition by T cells. Experimental evidence has been obtained for five different interactions between the T cell receptor (TCR), the metal ion (highlighted in red) and the peptide presented at the surface of the major histocompatibility complex (MHC). (**A**) The metal ion haptenizes the presented peptide. Loading can occur directly from the extracellular space (a) or by transfer from a metal-binding protein [232] (b). The metal ion may also be pre-loaded to the MHC-presented peptide, e.g., by antigen-processing of a metal-binding protein (c). (**B**) Metal ion binding at the TCR complementarity-determining region 3 (CDR3). The metal ion may bind to the TCR CDR3. Recently, frequent Ni^2+^ binding via a histidine in the CDR3 of TCR α- or β-chain has been shown [115]. (**C**) Metal ion binding to conserved residues at the TCR-peptide-MHC interface. Ni^2+^ ions often bind via tyrosine_36_ in the CDR1 of TRAV9-2+ TCR and histidine_81_ in the MHC II β-chain [114,115]. (**D**) Recognition of cryptic epitopes. Metal ions may alter antigen-processing of a self-protein which in turn leads to the presentation of a metal-free cryptic self-peptide. (**E**) Metal ions may bind within the cleft between MHC and antigen peptide. The TCR is not activated by a self-pMHC complex (1). The metal ion binding leads to structural conformation changes of the presented peptide and creates a metal-free neo antigen recognized by the TCR (2). This type of interaction has been shown for beryllium (II) ions in combination with HLA-DP2 [233].

Non-metallic chemical allergens are considered to bind mainly covalently to proteins via electrophilic-nucleophilic interactions or via the formation of radicals. Alternatively, metal ions as well as some small chemicals bind via complex formation (coordinative binding) at the TCR-pMHC interface [7,230]. In chemical allergies, no pathogen is present. Therefore, self-proteins or peptides are altered to form chemically induced T cell epitopes. The majority of metal-specific T cells seems MHC restricted although exceptions have been reported [231]. Usually, due to the selection process in the thymus, T cells have a low affinity for self-pMHC complexes, which does not lead to activation. However, the presence of a metal ion at the TCR-pMHC interface or cryptic epitopes may provide enough interactions to exceed activation thresholds. The still limited data on individual metal-specific TCR mainly comprise CD4+ T cell clones since metal-reactivity is more abundant on this subset (see Section 4.1.2). Several mechanisms for the generation of metal-induced T cell epitopes have been proposed, which are supported by a varying amount of experimental evidence (Figure 4).

The activation of metal reactive T cells likely requires a metal-mediated bridge between the TCR and pMHC complex in most cases [7]. Metal ions can directly interact with endogenous peptides bound to the MHC (Figure 4A). The TCR can then recognize the metal ion which is present on the pMHC. Ni-binding to the amino acid histidine in an MHC II-presented peptide (CS325-341 EPSDKHIEQYLKKIKNS) has been shown by NMR spectroscopy (Figure 4A(a)). The “empty” peptide activated a CS325-341-specific TCR (clone HM.37), the addition of Ni^2+^ prevented this interaction [234]. Similarly, the addition of Au (III) inhibited the activation of a CST3 (CS378-398 DIEKKICKMEKCSSVFNVVNS)-specific TCR (clone BH26). In this latter case, the peptide does not contain a histidine and the position of Au^3+^ remains speculative [235]. Possibly, Au^3+^ stripped the peptide from the MHC II and this abolished TCR activation [236].

Metals may be transported in human skin and blood by Ni-binding co-mediators towards the vicinity of the MHC molecule. Thierse et al. showed that activation of Ni-specific CD4+ T cells via a Ni-saturated human serum albumin complex can be comparable to activation by metal salts at equimolar concentrations. The human serum albumin was not used as an antigen on the MHC but only served to transfer Ni ions (Figure 4A(b)) [232].

The metal ion may also bind to proteins that undergo antigen processing. Fixation of APC with, e.g., glutaraldehyde, has been used by several groups to distinguish extracellular metal ion binding from intracellular processing of metal-binding proteins (Figure 4A(c)). Moulon et al. studied 42 CD4+ MHC II-restricted T cell clones. They showed that 40% of the clones were unable to recognize Ni^2+^ on the surface of fixed APC [188]. If the epitope is stable after fixation, the metal recognition can be classified as processing-independent and, at the time, the formation of cryptic epitopes can be excluded. Regarding the processing-dependent epitopes, another possible explanation for the lack of T cell activation by fixed APC is the possibility that the conformation of the metal-binding pMHC has been altered and thus external metal ion binding is disturbed. This cannot be ruled out, as no controls were implemented. One example for a CD4+ TCR that recognizes Ni bound to an MHC II-presented peptide is clone “ANi-2.3” (Figure 3A) which reacts to a complex of Ni and an unknown peptide generated in human B cells but not in other cells, e.g., PBMC (Figure 4A(c)) [113]. For clone Ani-2.3, a mimotope was found, that can replace Ni along with the peptide on the surface of MHC. Ni was replaced by the p7 lysine. Antigen recognition of the mimotope and the original Ni-epitope was equally affected when the Ani2.3 TCR was mutated. A CDR3β D95 E mutation abolished recognition, indicating that a geometrically highly defined coordination complex rather than just a negative charged amino acid is required at that position [209]. The exact Ni-binding geometry, however, remains unclear since no structure containing Ni is available. Ani2.3 is already activated by small metal salt concentrations. The concentration required for Ni-induced TCR interactions varies widely as does their dependency on the presence of certain antigenic peptides [115]. Some Ni-specific TCR are activated by very low Ni concentrations, for example by pulsed and washed APC, or even in the absence of professional APC [115,237].

Ni^2+^ may not only be bound by the MHC-presented peptide but also by the TCR itself as recently shown by our group (Figure 4B). Adapting a CD154 upregulation assay to isolate Ni-specific CD4+ T cells, we were able to comprehensively analyze the Ni-reactive TCR repertoire by high-throughput sequencing [115]. In the amino acid composition of the CDR3 of TCR α- and β-chains, a histidine was particularly abundant among Ni-specific TCR (14% of α-chain CDR3 and 29% of β-chain CDR3 at 200 µM NiSO_4_). As shown for some example clones, one histidine in either TCR α- or β-chain was sufficient for Ni recognition. Ni-binding to the histidine in the CDR3 may exceed the activation threshold of a non-optimal self TCR-pMHC connection (Figure 4B).

Another major mechanism of Ni recognition was experimentally proven [114,115]. Ni forms a complex with tyrosine_36_ (IMGT nomenclature) in the CDR1 α-chain of the gene segment TRAV9-2 and with histidine_81_ in the MHC II β-chain (Figure 4C). In this case, Ni may be needed as a direct link between the TCR and MHC, with the specified residues being in close proximity (Figure 3B) [114]. Our group showed that 35% of Ni-specific T cells express the variable gene segment TRAV9-2 while only 5% of random T cells contain this segment [115]. The identified TCR repertoire features, i.e., overrepresentation of segment TRAV9-2 or a CDR3 histidine, occurred among naïve and memory CD4+ T cells in allergic and non-allergic individuals. This reflects the high numbers of Ni-specific T cells that are present in non-allergic individuals. Little is known about the association with TCR gene segments for other metal-specific TCR. In a murine BALB/cAJcl metal allergy model, Ni-sensitized and Pd-challenged mice showed a high frequency of TCR with three distinct V-J segment combinations (TRAV7-5/TRAJ56, TRAV8 d-1/ TRAJ49 and TRAV5-1/TRAJ 37) [238]. Takeda et al. studied the pathogenic T cells responsible for Pd allergies. They found that in C57 BL/6 mice CD8+ T cells with the TRAV 7-2*02 segment increased significantly [134]. However, not only murine models but also human TCR repertoires differ, so additional data for humans are needed [105].

Metal ions may be able to alter the processing of self-antigens, causing T cells to respond to cryptic self-peptides. Griem et al. investigated two bovine RNase-specific T cell clones that only reacted upon Au^3+^ pre-treatment but not to the native protein [239]. The addition of Pd^2+^ had the same effect while Ni^2+^ was ineffective. The cellular processing of the proteins modified by metals caused the presentation of metal-free cryptic self-peptides, which could then be recognized by T cells. The altering of the original protein with Au^3+^ and Pd^2+^ results in the same cryptic peptides, which could be a cause for metal-associated TCR cross-reactivity (Figure 4D) [240].

So far, no HLA haplotype associations have been identified for metal allergies except for Be which is linked to HLA-DP2 [233]. Be^2+^ engages in a binding mechanism in which the Be^2+^ ion is not in direct contact with the TCR. Be^2+^ is buried between glutamic acid_69_ in the MHC II β-chain and the presented peptides and causes conformational and biophysical changes on the surface of the complex, generating neoantigens (Figure 4E) [208]. HLA-independent activation has also been described, but only for two Ni-specific CD8+ TCR from two donors. Both clones proliferated even when Ni was presented on allogeneic B cells [231,241].

Cross-reactivity is conceivable for most of the mechanisms presented above since one metal ion may be replaced by another metal with the same ion charge and similar binding properties.

### 5.3. TCR Cross-Reactivity to Ni, Co, and Pd

TCR cross-reactivity to different metal ions has been investigated in vivo by patch testing and in vitro by restimulation of metal-specific T cell clones with different metal allergens. Regarding the former, various epidemiological patch testing studies from different timeframes and worldwide locations show that Ni-allergic individuals often react to Co^2+^ and Pd^2+^ similarly. Depending on the study, between 2.3 and 72% of Ni-allergic patients also react to Co^2+^ with a positive patch test reaction [82,242,243]. Between 11 and 40% of Ni-reactive individuals respond to Pd^2+^ [87,242,243]. In several studies, up to 83% co-sensitization of Co-allergic patients to Ni^2+^ is also observed in combination with other metals [244,245]. Up to 95% of Pd-reactive individuals co-react to Ni^2+^ [246,247]. Furthermore, 56% of Pd-allergic individuals react to Co^2+^ (vice versa [87]).

A study by Hindsen et al. showed flare-up reactions after oral Ni salt administration at the sites of previous ACD to Pd and, to a lesser extent, ACD to Co, indicating the existence of TCR cross-reactivity, although the extent at the individual T cell level remains to be determined [171]. In a murine metal allergy model, Ni-and Pd-sensitized mice showed an allergic response after challenge with Ni^2+^ and Pd^2+^ but not with Co^2+^, chromium (II), copper (II) or Au (II). When the Ni-sensitized mice were challenged with Ni^2+^ and Pd^2+^ simultaneously, the reaction appeared to be additive when compared with the response to each metal alone, indicating Ni-Pd-cross-reactivity [248].

The wide variation in the frequencies of co-sensitized patients between different metal ions indicates that it is difficult to investigate the extend of T cell cross-reactivity by patch testing only. One issue in analyzing cross-reactivity is that it is not known whether the analyzed patients have a history of immunologically relevant co-exposure, i.e., co-sensitization but not cross-reactivity is observed by patch testing. Additionally, the reliability of the patch test is limited, given the low reproducibility and the influence of different metal salts including the used concentrations and varying skin penetration capacities as well as reading protocols for results (See Section 4.2.1).

In vitro cross-reactivity assays may be a more reliable alternative to patch tests. Metal-specific T cell clones can be tested directly with different metals. However, a strong concentration-dependent activation of T cells has been observed [115,132,180]. Therefore, results regarding cross-reactivity should also be interpreted with respect to the metal salt concentrations used. In addition, graded responses may occur, e.g., a distinctive but lower or stronger activation to a cross-reactive metal ion at equimolar concentrations. The high variability between individual T cell clones from one donor and large interindividual donor differences complicate the interpretation of the limited data on T cell cross-reactivity. Given the high frequency of metal-specific T cells in non-allergic patients, analyzed clones may not be allergy-relevant. As a result, comprehensive testing of T cell clones is very time-consuming and has thus been not accomplished. Taking advantage of the CD154+ upregulation assay could speed up this process [115]. Cross-reactivity becomes likely with elements that are listed in close proximity in the PSE. Since Ni^2+^ and Pd^2+^ have physiochemical similarities, they can form similar complexes and thus trigger the same modifications in proteins present in the skin or pMHC. These could then in turn be recognized by the same T cells [249].

Until today, only a few Ni-specific T cell clones were tested for cross-reactivity to Co^2+^ and Pd^2+^ [188,250]. Most of the Ni-specific T cell clones were either specific for Ni^2+^ or cross-reactive only to Pd^2+^. Moulon et al. and Pistoor et al. each identified only one Ni-specific clone (4.13 and PPN.53), which cross-reacted to Pd^2+^ and to Co^2+^. To date, for Co-specific T cells, no cross-reactivity to Ni^2+^ could be found [251,252]. Moulon et al. also assessed the cross-reactivity of Pd-specific T cells. Pd-specific clones cross-reacted only to Ni^2+^ and cross-reactivity varied depending on the donor. In one particular donor, all six clones examined, which were established by limited dilution from a line, also responded to Ni^2+^. In the second donor studied, only one of three clones reacted positively [188]. Cross-reactive T cells showed the same MHC restriction as with the original antigen, indicating exchange of the metal ion at the original epitope site [250].

Ni-specific naïve CD4+ and CD8+ T cells also showed a low rate of cross-reactivity to Co^2+^. Out of 11 Ni-specific CD4+ naïve T cells, only twocross-reacted to Co^2+^. Among four Ni-specific CD8+ naïve T cells, no cross-reactivity could be observed [180].

In summary, available experimental results indicate some cross-reactivity among Ni-, Co- and Pd-specific TCR but the exact percentages of cross-reactive T cells and details of the molecular recognition mechanisms remain unclear.

### 5.4. TCR Cross-Reactivity between Other Metal Allergens

Only very few Ni-specific CD4+ T cell clones have been investigated for cross-reactivity to other metal ions than Co^2+^ and Pd^2+^ and a minority of these proved cross-reactive to various metal ions. These metal ions could utilize the same binding mechanism as Ni^2+^, by coordinating similarly with the pMHC [113]. The Ni-specific TCR (ANi-2.3) cross-reacted to copper (II) and Au^2+^. In addition to Ni-Pd cross-reactivity, Moulon et al. and Pistoor et al. found Ni-specific T cell clones that cross-reacted solely with copper (II) but not with chromium (VI), Co^2+^ or Pd^2+^ [188,250]. The Ni-specific clones 4.13 and PPN.53, which cross-reacted to Co^2+^ and Pd^2+^, also reacted to copper(II) [188].

In summary, to date there has been only anecdotal in vitro research on the cross-reactivity of metal-specific T cells.

## 6. Conclusions

Much progress has been made in the understanding of the immunotoxic effects of metals. While some heavy metals like Ni [26] and Co [38] play an important role in biochemical and physiological processes in bacteria or animals, inadequate exposure can constitute a serious risk for human health. One of the most common forms of chronic immunotoxicity in humans is ACD. Adverse effects of metal ions that support the sensitization phase may be mediated by ROS [19] or protein binding. For example, Ni^2+^, Co^2+^ and Pd^2+^ induce pro-inflammatory gene expression by binding to the human TLR4 receptor [120]. Metal ions form complexes with proteins often via the imidazole moiety of histidine. The binding of metal ions to proteins (haptenization) is of fundamental importance for the development of allergic reactions, as it leads to the formation of allergen-induced T cell epitopes [7].

Although many details of the molecular interactions remain to be resolved, pioneering studies shed light on the way metal ions interact with the TCR-pMHC interface. Several binding mechanisms have been discovered, often using Ni^2+^ as a model allergen. New methods such as activation-induced marker assays combined with high-throughput TCR sequencing may allow a more quantitative analysis of metal-reactive T cell subpopulations as well as the deciphering of common metal-specific TCR repertoire features [115]. The complexation of Ni^2+^ via tyrosine 36 in the CDR1 of segment TRAV9-2 and histidine 81 in the MHC β-chain by a large fraction of TCR among Ni-specific CD4+ T cells was shown [114,115]. In addition, our group discovered that Ni^2+^ may not only bind to the surface of the pMHC complex via various pathways but that it is often complexed by histidine residues in the CDR3 of αβ TCR from CD4+ T cells [115].

Cross-reactivity to peptide antigens was first postulated in 1977 [198] and seems to be ubiquitous, because the huge human αβ TCR (≥10^8^ [204]) is still dwarfed by the number of possible pMHC complexes [202]. The mechanism of cross-reactivity is mainly based on conserved binding sites between TCR and pMHC [213], as well as on induced-fit [224], with no clear separation between both concepts. The diagnostic standard for ACD to date is the patch test, which has clear disadvantages such as limited reproducibility, especially for metal allergens [166]. The patch test is also rather unsuitable for the investigation of cross-reactivity since co-sensitization may be due to unknown previous co-exposure and different skin penetration capacities of the investigated metal salts confound results. In vitro tests could overcome some of the limitations of patch testing. In case of metal allergies, the use of diagnostic in vitro tests is currently limited due to the high frequency of metal-specific T cells in non-allergic individuals and the lack of a possibility to identify the pathogenic populations. Regarding cross-reactivity, to date, there are only anecdotal data from a limited number of donors on the cross-reactivity of Ni-specific T cell clones [188,250]. They point towards a cross-reactivity of Ni-specific T cells to Pd^2+^ and more rarely to Co^2+^ for CD4+ T cells. Here, clearly more research is needed.

The knowledge about the cross-reactivity pattern of metal-specific TCR as the foundation of the adaptive immune response may not only be important for the choice of implant materials to avoid incompatibility reactions. It could also influence the regulation of metal content in consumer products. Nevertheless, the avoidance of allergens, e.g., by a switch to alternative materials such as ceramics for implants is the most effective prevention for the elicitation of metal allergies. Therefore, it is crucial to obtain a deeper mechanistic understanding of the TCR interactions with metal-induced T cell epitopes and the underlying TCR repertoire features. In this context, the in vivo relevance of mechanisms and metal-specific T cells identified in vitro should be further elucidated. Finally, to obtain a deeper insight into ACD, the penetration of metal ions into the skin and the role of antigen-specific T cell subpopulations including T_RM_ should be assessed.

## Data Availability

Not applicable.

## Abbreviations

ACD: allergic contact dermatitis; Au, gold; Be, beryllium; CDR, complementarity-determining regions; CMV, cytomegalovirus; Co, Co^2+^ ions, cobalt; ELISpot, Enzyme-linked-immuno-Spot Assay; HLA, human leukocyte antigen; ICP-MS, inductively coupled plasma mass spectrometry; IFN, interferon; IL, interleukin; MHC, major histocompatibility complex; MW, molecular weight; Ni, Ni^2+^ ions, nickel; PBMC, peripheral blood mononuclear cells; Pd, Pd^2+^ ions, palladium; pMHC, peptide-MHC; PRR, pattern recognition receptors; PSE, periodic table of elements; REACH, Registration, Evaluation, Authorization and Restriction of Chemicals; ROS, reactive oxygen species; TCR, T cell receptor; T_H_, T helper cells; TLR, Toll-like receptor; T_RM,_ tissue-resident memory T cells.

## References

[1] Alinaghi F., Bennike N.H., Egeberg A., Thyssen J.P., Johansen J.D. (2019). Prevalence of contact allergy in the general population: A systematic review and meta-analysis. Contact Dermat..

[2] Thyssen J.P., Menné T. (2010). Metal Allergy—A Review on Exposures, Penetration, Genetics, Prevalence, and Clinical Implications. Chem. Res. Toxicol..

[3] Nguyen A.V., Soulika A.M. (2019). The Dynamics of the Skin’s Immune System. Int. J. Mol. Sci..

[4] Gittler J.K., Krueger J.G., Guttman-Yassky E. (2013). Atopic dermatitis results in intrinsic barrier and immune abnormalities: Implications for contact dermatitis. J. Allergy Clin. Immunol..

[5] Uter W., Werfel T., Lepoittevin J.-P., White I.R. (2020). Contact Allergy—Emerging Allergens and Public Health Impact. Int. J. Environ. Res. Public Health.

[6] Chen J.K., Thyssen J.P. (2018). Metal Allergy: From Dermatitis to Implant and Device Failure.

[7] Thierse H.-J., Gamerdinger K., Junkes C., Guerreiro N., Weltzien H.U. (2005). T cell receptor (TCR) interaction with haptens: Metal ions as non-classical haptens. Toxicology.

[8] Schmidt M., Goebeler M. (2015). Immunology of metal allergies. J. Dtsch. Dermatol. Ges..

[9] Azeem M., Kader H., Kerstan A., Hetta H.F., Serfling E., Goebeler M., Muhammad K. (2020). Intricate Relationship Between Adaptive and Innate Immune System in Allergic Contact Dermatitis. Yale J. Biol. Med..

[10] Mehri A. (2020). Trace Elements in Human Nutrition (II)—An Update. Int. J. Prev. Med..

[11] Masterton W.L., Hurley C.N. (2015). Chemistry: Principles and Reactions.

[12] Tchounwou P.B., Yedjou C.G., Patlolla A.K., Sutton D.J. (2012). Heavy Metal Toxicity and the Environment. Mol. Clin. Environ. Toxicol..

[13] Römpp R., Blaß W. (2016). “Schwermetalle”. Thieme Gruppe. https://roempp.thieme.de/lexicon/RD-19-01472..

[14] Shahzad B., Tanveer M., Rehman A., Alam Cheema S., Fahad S., Rehman S., Sharma A. (2018). Nickel; Whether toxic or essential for plants and environment—A review. Plant Physiol. Biochem..

[15] Crans D.C., Kostenkova K. (2020). Open questions on the biological roles of first-row transition metals. Commun. Chem..

[16] Sigel H., Sigel A. (2019). The bio-relevant metals of the periodic table of the elements. Zeitschrift für Naturforschung B.

[17] Jaishankar M., Tseten T., Anbalagan N., Mathew B.B., Beeregowda K.N. (2014). Toxicity, mechanism and health effects of some heavy metals. Interdiscip. Toxicol..

[18] Renu K., Chakraborty R., Myakala H., Koti R., Famurewa A.C., Madhyastha H., Vellingiri B., George A., Gopalakrishnan A.V. (2021). Molecular mechanism of heavy metals (Lead, Chromium, Arsenic, Mercury, Nickel and Cadmium)—induced hepatotoxicity—A review. Chemosphere.

[19] Bjørklund G., Dadar M., Chirumbolo S., Aaseth J., Peana M.F. (2020). Metals, autoimmunity, and neuroendocrinology: Is there a connection?. Environ. Res..

[20] Heddle J., Scott D., Unzai S., Park S.-Y., Tame J.R.H. (2003). Crystal Structures of the Liganded and Unliganded Nickel-binding Protein NikA from Escherichia coli. J. Biol. Chem..

[21] Dudev M., Wang J., Dudev T., Lim C. (2006). Factors Governing the Metal Coordination Number in Metal Complexes from Cambridge Structural Database Analyses. J. Phys. Chem. B.

[22] Halcrow M., Christou G. (1994). Biomimetic Chemistry of Nickel. Chem. Rev..

[23] Sundberg R.J., Martin R.B. (1974). Interactions of histidine and other imidazole derivatives with transition metal ions in chemical and biological systems. Chem. Rev..

[24] Fernandes H.S., Teixeira C.S.S., Sousa S.F., Cerqueira N.M.F.S.A. (2019). Formation of Unstable and very Reactive Chemical Species Catalyzed by Metalloenzymes: A Mechanistic Overview. Molecules.

[25] Zhang Y., Zheng J. (2020). Bioinformatics of Metalloproteins and Metalloproteomes. Molecules.

[26] Nim Y.S., Wong K.-B. (2019). The Maturation Pathway of Nickel Urease. Inorganics.

[27] Zambelli B., Banaszak K., Merloni A., Kiliszek A., Rypniewski W., Ciurli S. (2013). Selectivity of Ni(II) and Zn(II) binding to Sporosarcina pasteurii UreE, a metallochaperone in the urease assembly: A calorimetric and crystallographic study. JBIC J. Biol. Inorg. Chem..

[28] Boer J.L., Mulrooney S.B., Hausinger R.P. (2014). Nickel-dependent metalloenzymes. Arch. Biochem. Biophys..

[29] Genchi G., Carocci A., Lauria G., Sinicropi M.S., Catalano A. (2020). Nickel: Human Health and Environmental Toxicology. Int. J. Environ. Res. Public Health.

[30] Chen H., Giri N., Zhang R., Yamane K., Zhang Y., Maroney M., Costa M. (2010). Nickel Ions Inhibit Histone Demethylase JMJD1A and DNA Repair Enzyme ABH2 by Replacing the Ferrous Iron in the Catalytic Centers. J. Biol. Chem..

[31] Canaz E., Kilinc M., Sayar H., Kiran G., Ozyurek E. (2017). Lead, selenium and nickel concentrations in epithelial ovarian cancer, borderline ovarian tumor and healthy ovarian tissues. J. Trace Elem. Med. Biol..

[32] Guo H., Liu H., Jian Z., Cui H., Fang J., Zuo Z., Deng J., Li Y., Wang X., Zhao L. (2020). Immunotoxicity of nickel: Pathological and toxicological effects. Ecotoxicol. Environ. Saf..

[33] Saini S., Nair N., Saini M.R. (2013). Embryotoxic and Teratogenic Effects of Nickel in Swiss Albino Mice during Organogenetic Period. BioMed Res. Int..

[34] Leonard A., Jacquet P. (1984). Embryotoxicity and genotoxicity of nickel. IARC Sci. Publ..

[35] Khrustalev V.V., Khrustaleva T.A., Poboinev V.V., Karchevskaya C.I., Shablovskaya E.A., Terechova T.G. (2019). Cobalt(ii) cation binding by proteins. Metallomics.

[36] Kumar S., Gupta R. (2017). Cobalt Complexes Catalyze Reduction of Nitro Compounds: Mechanistic Studies. Chemistryselect.

[37] Kobayashi M., Shimizu S. (1999). Cobalt proteins. JBIC J. Biol. Inorg. Chem..

[38] Simonsen L.O., Harbak H., Bennekou P. (2012). Cobalt metabolism and toxicology—A brief update. Sci. Total. Environ..

[39] Wahlqvist F., Bryngelsson I.-L., Westberg H., Vihlborg P., Andersson L. (2020). Dermal and inhalable cobalt exposure—Uptake of cobalt for workers at Swedish hard metal plants. PLoS ONE.

[40] Lauwerys R., Lison D. (1994). Health risks associated with cobalt exposure—An overview. Sci. Total. Environ..

[41] Green B., Griffiths E., Almond S. (2017). Neuropsychiatric symptoms following metal-on-metal implant failure with cobalt and chromium toxicity. BMC Psychiatry.

[42] Leyssens L., Vinck B., Van Der Straeten C., Dhooge I., Wuyts F.L., Maes L.K. (2021). The ototoxic potential of cobalt from metal-on-metal hip implants: A pilot study on the patient-reported auditory, vestibular, and general neurological outcome. Int. J. Audiol..

[43] Leyssens L., Vinck B., Van Der Straeten C., Wuyts F., Maes L. (2017). Cobalt toxicity in humans—A review of the potential sources and systemic health effects. Toxicology.

[44] Sun X., Yu G., Xu Q., Li N., Xiao C., Yin X., Cao K., Han J., He Q.-Y. (2013). Putative cobalt- and nickel-binding proteins and motifs in Streptococcus pneumoniae. Metallomics.

[45] Yang J., Black J. (1994). Competitive binding of chromium, cobalt and nickel to serum proteins. Biomaterials.

[46] Merget R., Rosner G. (2001). Evaluation of the health risk of platinum group metals emitted from automotive catalytic converters. Sci. Total. Environ..

[47] Jbara M., Maity S.K., Brik A. (2017). Palladium in the Chemical Synthesis and Modification of Proteins. Angew. Chem. Int. Ed..

[48] Faurschou A., Menné T., Johansen J.D., Thyssen J.P. (2011). Metal allergen of the 21st century-a review on exposure, epidemiology and clinical manifestations of palladium allergy. Contact Dermat..

[49] Kielhorn J., Melber C., Keller D., Mangelsdorf I. (2002). Palladium—A review of exposure and effects to human health. Int. J. Hyg. Environ. Health.

[50] Yoshida S., Sakamoto H., Mikami H., Onuma K., Shoji T., Nakagawa H., Hasegawa H., Amayasu H. (1999). Palladium allergy exacerbating bronchial asthma. J. Allergy Clin. Immunol..

[51] Gebel T., Lantzsch H., Pleßow K., Dunkelberg H. (1997). Genotoxicity of platinum and palladium compounds in human and bacterial cells. Mutat. Res. Toxicol. Environ. Mutagen..

[52] Roach K.A., Stefaniak A.B., Roberts J.R. (2019). Metal nanomaterials: Immune effects and implications of physicochemical properties on sensitization, elicitation, and exacerbation of allergic disease. J. Immunotoxicol..

[53] Anderson S.E., Meade B.J. (2014). Potential Health Effects Associated with Dermal Exposure to Occupational Chemicals. Environ. Health Insights.

[54] Teo W.Z.W., Schalock P.C. (2017). Metal Hypersensitivity Reactions to Orthopedic Implants. Dermatol. Ther..

[55] Haddad S.F., Helm M.M., Meath B., Adams C., Packianathan N., Uhl R. (2019). Exploring the Incidence, Implications, and Relevance of Metal Allergy to Orthopaedic Surgeons. JAAOS Glob. Res. Rev..

[56] Li T., Wan Y., Ben Y., Fan S., Hu J. (2017). Relative importance of different exposure routes of heavy metals for humans living near a municipal solid waste incinerator. Environ. Pollut..

[57] Nag R., O’Rourke S.M., Cummins E. (2022). Risk factors and assessment strategies for the evaluation of human or environmental risk from metal(loid)s—A focus on Ireland. Sci. Total Environ..

[58] Ahlström M.G., Thyssen J.P., Wennervaldt M., Menné T., Johansen J.D. (2019). Nickel allergy and allergic contact dermatitis: A clinical review of immunology, epidemiology, exposure, and treatment. Contact Dermat..

[59] Katta R., Schlichte M. (2014). Diet and Dermatitis: Food Triggers. J. Clin. Aesthetic Dermatol..

[60] Salik E., Løvik I., Andersen K.E., Bygum A. (2016). Persistent Skin Reactions and Aluminium Hypersensitivity Induced by Childhood Vaccines. Acta Derm. Venereol..

[61] Ścibior A., Pietrzyk Ł., Plewa Z., Skiba A. (2020). Vanadium: Risks and possible benefits in the light of a comprehensive overview of its pharmacotoxicological mechanisms and multi-applications with a summary of further research trends. J. Trace Elem. Med. Biol..

[62] Diepgen T.L., Ofenloch R., Bruze M., Bertuccio P., Cazzaniga S., Coenraads P.-J., Elsner P., Goncalo M., Svensson Å., Naldi L. (2016). Prevalence of contact allergy in the general population in different European regions. Br. J. Dermatol..

[63] Mortz C.G., Lauritsen J.M., Bindslev-Jensen C., Andersen K.E. (2002). Nickel Sensitization in Adolescents and Association with Ear Piercing, Use of Dental Braces and Hand Eczema. Acta Derm. Venereol..

[64] McSweeney S.M., White I.R., Kimber I., McFadden J.P., Tziotzios C. (2020). Contact allergy across the human lifespan. J. Allergy Clin. Immunol..

[65] Li L.-F. (2010). Contact sensitization to European baseline series of allergens in university students in Beijing. Contact Dermat..

[66] Zhao L., Li L.-F. (2015). Contact sensitization to 34 common contact allergens in university students in Beijing. Contact Dermat..

[67] DeKoven J.G., Warshaw E.M., Zug K.A., Maibach H.I., Belsito D.V., Sasseville D., Taylor J.S., Fowler J.F., Mathias C.G.T., Marks J.G. (2018). North American Contact Dermatitis Group Patch Test Results: 2015–2016. Dermatitis.

[68] Filatova D., Cherpak C. (2020). Mechanisms of Nickel-Induced Cell Damage in Allergic Contact Dermatitis and Nutritional Intervention Strategies. Endocr. Metab. Immune Disord.—Drug Targets.

[69] Thyssen J.P., Linneberg A., Menné T., Johansen J.D. (2007). The epidemiology of contact allergy in the general population—Prevalence and main findings. Contact Dermat..

[70] Uter W., Bauer A., Fortina A.B., Bircher A.J., Brans R., Buhl T., Cooper S.M., Czarnecka-Operacz M., Dickel H., Dugonik A. (2021). Patch test results with the European baseline series and additions thereof in the ESSCA network, 2015–2018. Contact Dermat..

[71] Silverberg N.B., Pelletier J.L., Jacob S.E., Schneider L.C. (2020). Nickel Allergic Contact Dermatitis: Identification, Treatment, and Prevention. Pediatrics.

[72] Fischer L.A., Menné T., Johansen J.D. (2007). Dose per unit area—A study of elicitation of nickel allergy. Contact Dermat..

[73] Ahlström M.G., Midander K., Menné T., Lidén C., Johansen J.D., Julander A., Thyssen J.P. (2019). Nickel deposition and penetration into the stratum corneum after short metallic nickel contact: An experimental study. Contact Dermat..

[74] Kasper-Sonnenberg M., Sugiri D., Wurzler S., Ranft U., Dickel H., Wittsiepe J., Hölzer J., Lemm F., Eberwein G., Altmeyer P. (2011). Prevalence of nickel sensitization and urinary nickel content of children are increased by nickel in ambient air. Environ. Res..

[75] European Parliament (2004). Commission Directive 2004/96/EC of 27 September 2004 amending Council Directive 76/769/EEC as regards restrictions on the marketing and use of nickel for piercing post assemblies for purpose of adapting its Annex I to technical progress. Off. J. Eur. Union.

[76] European Parliament (1994). European Parliament and Council Directive 94/27/EC of 30 June 1994: Amending for the 12th time Directive 76/769/EEC on the approximation of the laws, regulations and administrative provisions of the Member States relating to restrictions on the marketing and use of certain dangerous substances and preparations. Off. J. Eur. Commun..

[77] European Parliament (2006). Regulation (EC) No 1907/2006 of the European Parliament and of the Council of 18 December 2006 concerning the registration, evaluation, authorisation and restriction of chemicals (REACH), establishing a European Chemicals Agency, amending Directive 1999/45/EC and repealing Council Regulation (EEC) No 793/93 and Commission Regulation (EC) No 1488/94 as well as Council Directive 76/769/EEC and Commission Directives 91/155/EEC, 93/67/EEC, 93/105/EC and 2000/21/EC. Off. J. Eur. Union.

[78] Ahlström M.G., Menné T., Thyssen J.P., Johansen J.D. (2017). The European nickel regulation and changes since its introduction. Contact Dermat..

[79] Thierse H.-J., Luch A. (2019). Verbraucherschutz und Risikobewertung—Allergieauslösende Substanzen in Verbraucherprodukten. Allergo J..

[80] European Parliament (2004). Directive 2004/107/EC of the European Parliament and of the Council of 15 December 2004 relating to arsenic, cadmium, mercury, nickel and polycyclic aromatic hydrocarbons in ambient air. Off. J. Eur. Union.

[81] Erfani B., Lidén C., Midander K. (2015). Short and frequent skin contact with nickel. Contact Dermat..

[82] Hegewald J., Uter W., Pfahlberg A., Geier J., Schnuch A., Ivdk T. (2005). A multifactorial analysis of concurrent patch-test reactions to nickel, cobalt, and chromate. Allergy.

[83] Forte G., Petrucci F., Bocca B. (2008). Metal allergens of growing significance: Epidemiology, immunotoxicology, strategies for testing and prevention. Inflamm. Allergy—Drug Targets.

[84] Rubins A., Romanova A., Septe M., Maddukuri S., Schwartz R.A., Rubins S. (2020). Contact dermatitis: Etiologies of the allergic and irritant type. Acta Dermatovenerol. Alp. Pannonica et Adriat..

[85] European Parliament (2019). Commission Delegated Regulation (EU) 2020/217 of 4 October 2019 amending, for the purposes of its adaptation to technical and scientific progress, Regulation (EC) No 1272/2008 of the European Parliament and of the Council on classification, labelling and packaging of substances and mixtures and correcting that Regulation. Off. J. Eur. Union.

[86] European Chemicals Agency (2020). Opinion on an Annex XV Dossier Proposing Restrictions on Skin Sensitising Substances.

[87] González-Ruiz L., De Caso E.V., Peña-Sánchez R., Silvestre-Salvador J.F., Peña-Sáncez R. (2019). Delayed hypersensitivity to palladium dichloride: 15-year retrospective study in a skin allergy unit. Contact Dermat..

[88] Durosaro O., el-Azhary R.A. (2009). A 10-year retrospective study on palladium sensitivity. Dermatitis.

[89] Kapp F., Summer B., Thomas P. (2020). Usefulness of lymphocyte transformation test and in vitro cytokine release in differentiating between independent and cross-reacting nickel/palladium allergy. Immun. Inflamm. Dis..

[90] Nucera E., Chini R., Rizzi A., Schiavino D., Buonomo A., Aruanno A., Ricci R., Mangiola F., Campanale M.C., Gasbarrini A. (2019). Eosinophilic oesophagitis (in nickel-allergic patient) regressed after nickel oral desensitization: A case report. Int. J. Immunopathol. Pharmacol..

[91] Marcant P., Alcaraz I., Beauval N., Martin de Lassalle E., Chantelot C., Staumont-Sallé D. (2021). Metal implant allergy: A diagnostic challenge illustrating the limits of the nickel spot test. Contact Dermat..

[92] Van Der Bent S.A., Berg T., Karst U., Sperling M., Rustemeyer T. (2019). Allergic reaction to a green tattoo with nickel as a possible allergen. Contact Dermat..

[93] Weiß K.T., Schreiver I., Siewert K., Luch A., Haslböck B., Berneburg M., Bäumler W. (2021). Tattoos–more than just colored skin? Searching for tattoo allergens. JDDG J. Dtsch. Dermatol. Ges..

[94] Simonsen A.B., Friis U.F., Johansen J.D., Zachariae C., Sloth J.J., Thyssen J.P. (2019). Occupational allergic contact dermatitis caused by cobalt in machine oil. Contact Dermat..

[95] Marsidi N., Beijnen J.H., Van Zuuren E.J. (2018). Palladium-induced granulomas analysed with inductively coupled plasma mass spectrometry. Contact Dermat..

[96] Kaplan D.H., Igyártó B.Z., Gaspari A.A. (2012). Early immune events in the induction of allergic contact dermatitis. Nat. Rev. Immunol..

[97] Helou D.G., Martin S.F., Pallardy M., Chollet-Martin S., Kerdine-Römer S. (2019). Nrf2 Involvement in Chemical-Induced Skin Innate Immunity. Front. Immunol..

[98] Fitzpatrick J.M., Roberts D.W., Patlewicz G. (2017). Is skin penetration a determining factor in skin sensitization potential and potency? Refuting the notion of a LogKow threshold for skin sensitization. J. Appl. Toxicol..

[99] Ouchi T., Kubo A., Yokouchi M., Adachi T., Kobayashi T., Kitashima D.Y., Fujii H., Clausen B., Koyasu S., Amagai M. (2011). Langerhans cell antigen capture through tight junctions confers preemptive immunity in experimental staphylococcal scalded skin syndrome. J. Exp. Med..

[100] Kabashima K., Honda T., Ginhoux F., Egawa G. (2019). The immunological anatomy of the skin. Nat. Rev. Immunol..

[101] Jakob T., Ring J., Udey M.C. (2001). Multistep navigation of Langerhans/ dendritic cells in and out of the skin. J. Allergy Clin. Immunol..

[102] Vargas P., Maiuri P., Bretou M., Sáez P.J., Pierobon P., Maurin M., Chabaud M., Lankar D., Obino D., Terriac E. (2016). Innate control of actin nucleation determines two distinct migration behaviours in dendritic cells. Nat. Cell Biol..

[103] E Macatonia S., Edwards A.J., Knight S.C. (1986). Dendritic cells and the initiation of contact sensitivity to fluorescein isothiocyanate. Immunology.

[104] Gaide O., Emerson R.O., Jiang X., Gulati N., Nizza S.T., Desmarais C., Robins H., Krueger J.G., Clark R.A., Kupper T.S. (2015). Common clonal origin of central and resident memory T cells following skin immunization. Nat. Med..

[105] Murata A., Hayashi S.-I. (2020). CD4+ Resident Memory T Cells Mediate Long-Term Local Skin Immune Memory of Contact Hypersensitivity in BALB/c Mice. Front. Immunol..

[106] Bonneville M., Chavagnac C., Vocanson M., Rozieres A., Benetiere J., Pernet I., Denis A., Nicolas J.-F., Hennino A. (2007). Skin Contact Irritation Conditions the Development and Severity of Allergic Contact Dermatitis. J. Investig. Dermatol..

[107] McKee A.S., Fontenot A.P. (2016). Interplay of innate and adaptive immunity in metal-induced hypersensitivity. Curr. Opin. Immunol..

[108] Li X., Zhong F. (2014). Nickel Induces Interleukin-1β Secretion via the NLRP3–ASC–Caspase-1 Pathway. Inflammation.

[109] Zheng D., Liwinski T., Elinav E. (2020). Inflammasome activation and regulation: Toward a better understanding of complex mechanisms. Cell Discov..

[110] Caicedo M.S., Desai R., McAllister K., Reddy A., Jacobs J.J., Hallab N.J. (2009). Soluble and particulate Co-Cr-Mo alloy implant metals activate the inflammasome danger signaling pathway in human macrophages: A novel mechanism for implant debris reactivity. J. Orthop. Res..

[111] Chamaon K., Schönfeld P., Awiszus F., Bertrand J., Lohmann C.H. (2019). Ionic cobalt but not metal particles induces ROS generation in immune cells in vitro. J. Biomed. Mater. Res. Part B Appl. Biomater..

[112] Ercal N., Gurer-Orhan H., Aykin-Burns N. (2001). Toxic Metals and Oxidative Stress Part I: Mechanisms Involved in Me-tal induced Oxidative Damage. Curr. Top. Med. Chem..

[113] Lu L., Vollmer J., Moulon C., Weltzien H.U., Marrack P., Kappler J. (2003). Components of the Ligand for a Ni++ Reactive Human T Cell Clone. J. Exp. Med..

[114] Gamerdinger K., Moulon C., Karp D.R., Van Bergen J., Koning F., Wild D., Pflugfelder U., Weltzien H.U. (2003). A New Type of Metal Recognition by Human T Cells: Contact residues for peptide-independent bridging of T cell receptor and major histocompatibility complex by nickel. J. Exp. Med..

[115] Aparicio-Soto M., Riedel F., Leddermann M., Bacher P., Scheffold A., Kuhl H., Timmermann B., Chudakov D.M., Molin S., Worm M. (2020). TCRs with segment TRAV9-2 or a CDR3 histidine are overrepresented among nickel-specific CD4+ T cells. Allergy.

[116] Höper T., Siewert K., Dumit V.I., von Bergen M., Schubert K., Haase A. (2021). The Contact Allergen NiSO4 Triggers a Distinct Molecular Response in Primary Human Dendritic Cells Compared to Bacterial LPS. Front. Immunol..

[117] Schmidt M., Raghavan B., Müller V., Vogl T., Fejer G., Tchaptchet S., Keck S., Kalis C., Nielsen P.J., Galanos C. (2010). Crucial role for human Toll-like receptor 4 in the development of contact allergy to nickel. Nat. Immunol..

[118] Vennegaard M.T., Dyring-Andersen B., Skov L., Nielsen M.M., Schmidt J.D., Bzorek M., Poulsen S.S., Thomsen A.R., Woetmann A., Thyssen J.P. (2014). Epicutaneous exposure to nickel induces nickel allergy in mice via a MyD88-dependent and interleukin-1-dependent pathway. Contact Dermat..

[119] Guo H., Liu H., Jian Z., Cui H., Fang J., Zuo Z., Deng J., Li Y., Wang X., Zhao L. (2019). Nickel induces inflammatory activation via NF-κB, MAPKs, IRF3 and NLRP3 inflammasome signaling pathways in macrophages. Aging.

[120] Rachmawati D., Bontkes H.J., Verstege M.I., Muris J., von Blomberg B.M.E., Scheper R.J., van Hoogstraten I.M.W. (2013). Transition metal sensing by Toll-like receptor-4: Next to nickel, cobalt and palladium are potent human dendritic cell stimulators. Contact Dermat..

[121] Jensen C.S., Lisby S., Baadsgaard O., Volund A., Menne T. (2002). Decrease in nickel sensitization in a Danish schoolgirl population with ears pierced after implementation of a nickel-exposure regulation. Br. J. Dermatol..

[122] Liu L., Zhong Q., Tian T., Dubin K., Athale S.K., Kupper T.S. (2010). Epidermal injury and infection during poxvirus immunization is crucial for the generation of highly protective T cell–mediated immunity. Nat. Med..

[123] Christiansen E.S., Andersen K.E., Bindslev-Jensen C., Halken S., Kjaer H.F., Eller E., Høst A., Mortz C.G. (2016). Low patch test reactivity to nickel in unselected adolescents tested repeatedly with nickel in infancy. Pediatr. Allergy Immunol..

[124] Brasch J., Becker D., Aberer W., Bircher A., Kränke B., Jung K., Przybilla B., Biedermann T., Werfel T., John S.M. (2014). Leitlinie Kontaktekzem. Allergo J..

[125] Martin S.F., Esser P.R. (2021). Innate Immune Mechanisms in Contact Dermatitis.

[126] Clark R.A. (2015). Resident memory T cells in human health and disease. Sci. Transl. Med..

[127] Gadsbøll A.-S.Ø., Jee M.H., Funch A.B., Alhede M., Mraz V., Weber J.F., Callender L.A., Carroll E.C., Bjarnsholt T., Woetmann A. (2020). Pathogenic CD8+ Epidermis-Resident Memory T Cells Displace Dendritic Epidermal T Cells in Allergic Dermatitis. J. Investig. Dermatol..

[128] Schmidt J.D., Ahlström M.G., Johansen J.D., Dyring-Andersen B., Agerbeck C., Nielsen M.M., Poulsen S.S., Woetmann A., Ødum N., Thomsen A.R. (2017). Rapid allergen-induced interleukin-17 and interferon-γ secretion by skin-resident memory CD8+T cells. Contact Dermat..

[129] Kish D.D., Li X., Fairchild R.L. (2009). CD8 T Cells Producing IL-17 and IFN-γ Initiate the Innate Immune Response Required for Responses to Antigen Skin Challenge. J. Immunol..

[130] Kapsenberg M.L., Res P., Bos J.D., Schootemijer A., Teunissen M.B.M., Van Schooten W. (1987). Nickel-specific T lymphocyte clones derived from allergic nickel-contact dermatitis lesions in man: Heterogeneity based on requirement of dendritic antigen-presenting cell subsets. Eur. J. Immunol..

[131] Gawkrodger D.J., Carr M.M., McVittie E., Guy K., Hunter J.A.A. (1987). Keratinocyte Expression of MHC Class II Antigens in Allergic Sensitization and Challenge Reactions and in Irritant Contact Dermatitis. J. Investig. Dermatol..

[132] Cavani A., Mei D., Corinti S., Girolomoni G., Guerra E., Giani M., Pirrotta L., Puddu P. (1998). Patients with Allergic Contact Dermatitis to Nickel and Nonallergic Individuals Display Different Nickel-Specific T Cell Responses. Evidence for the Presence of Effector CD8+ and Regulatory CD4+ T Cells. J. Investig. Dermatol..

[133] Kawano M., Nakayama M., Aoshima Y., Nakamura K., Ono M., Nishiya T., Nakamura S., Takeda Y., Dobashi A., Takahashi A. (2014). NKG2D+ IFN-γ+ CD8+ T Cells Are Responsible for Palladium Allergy. PLoS ONE.

[134] Takeda Y., Suto Y., Ito K., Hashimoto W., Nishiya T., Ueda K., Narushima T., Takahashi T., Ogasawara K. (2017). TRAV7-2*02 Expressing CD8+ T Cells Are Responsible for Palladium Allergy. Int. J. Mol. Sci..

[135] Vocanson M., Hennino A., Chavagnac C., Saint-Mezard P., Dubois B., Kaiserlian D., Nicolas J.-F. (2005). Contribution of CD4+and CD8+T-cells in contact hypersensitivity and allergic contact dermatitis. Expert Rev. Clin. Immunol..

[136] Cavani A., Nasorri F., Ottaviani C., Sebastiani S., De Pità O., Girolomoni G. (2003). Human CD25+ Regulatory T Cells Maintain Immune Tolerance to Nickel in Healthy, Nonallergic Individuals. J. Immunol..

[137] Moed H., Von Blomberg B.M.E., Bruynzeel D.P., Scheper R.J., Gibbs S., Rustemeyer T. (2005). Regulation of nickel-induced T-cell responsiveness by CD4+CD25+cells in contact allergic patients and healthy individuals. Contact Dermat..

[138] Wolfl M., Kuball J., Ho W.Y., Nguyen H., Manley T.J., Bleakley M., Greenberg P.D. (2007). Activation-induced expression of CD137 permits detection, isolation, and expansion of the full repertoire of CD8+ T cells responding to antigen without requiring knowledge of epitope specificities. Blood.

[139] Moed H., Boorsma D., Stoof T., Von Blomberg B., Bruynzeel D., Scheper R., Gibbs S., Rustemeyer T. (2004). Nickel-responding T cells are CD4+ CLA+ CD45RO+ and express chemokine receptors CXCR3, CCR4 and CCR10. Br. J. Dermatol..

[140] Sinigaglia F., Scheidegger D., Garotta G., Scheper R., Pletscher M., Lanzavecchia A. (1985). Isolation and characterization of Ni-specific T cell clones from patients with Ni-contact dermatitis. J. Immunol..

[141] Minang J.T., Areström I., Troye-Blomberg M., Lundeberg L., Ahlborg N. (2006). Nickel, cobalt, chromium, palladium and gold induce a mixed Th1- and Th2-type cytokine response in vitro in subjects with contact allergy to the respective metals. Clin. Exp. Immunol..

[142] Minang J.T., Troye-Blomberg M., Lundeberg L., Ahlborg N. (2005). Nickel Elicits Concomitant and Correlated in vitro Production of Th1-, Th2-Type and Regulatory Cytokines in Subjects with Contact Allergy to Nickel. Scand. J. Immunol..

[143] Minang J.T., Areström I., Zuber B., Jönsson G., Troye-Blomberg M., Ahlborg N. (2006). Nickel-induced IL-10 down-regulates Th1- but not Th2-type cytokine responses to the contact allergen nickel. Clin. Exp. Immunol..

[144] Muris J., Feilzer A.J., Kleverlaan C.J., Rustemeyer T., van Hoogstraten I.M.W., Scheper R.J., von Blomberg B.M.E. (2012). Palladium-induced Th2 cytokine responses reflect skin test reactivity. Allergy.

[145] Dhingra N., Shemer A., da Rosa J.C., Rozenblit M., Fuentes-Duculan J., Gittler J.K., Finney R., Czarnowicki T., Zheng X., Xu H. (2014). Molecular profiling of contact dermatitis skin identifies allergen-dependent differences in immune response. J. Allergy Clin. Immunol..

[146] Bordignon V., Palamara F., Cordiali-Fei P., Vento A., Aiello A., Picardo M., Ensoli F., Cristaudo A. (2008). Nickel, palladium and rhodium induced IFN-gamma and IL-10 production as assessed by in vitro ELISpot-analysis in contact dermatitis patients. BMC Immunol..

[147] Todberg T., Zachariae C., Krustrup D., Skov L. (2018). The effect of treatment with anti-interleukin-17 in patients with allergic contact dermatitis. Contact Dermat..

[148] Ruge I.F., Skov L., Zachariae C., Thyssen J.P. (2020). Dupilumab treatment in two patients with severe allergic contact dermatitis caused by sesquiterpene lactones. Contact Dermat..

[149] Brasch J., Becker D., Aberer W., Bircher A., Kränke B., Jung K., Przybilla B., Biedermann T., Werfel T., John S.M. (2014). Guideline contact dermatitis. Allergo J. Int..

[150] Bourke J., Coulson I., English J. (2009). Guidelines for the management of contact dermatitis: An update. Br. J. Dermatol..

[151] Johansen J.D., Aalto-Korte K., Agner T., Andersen K.E., Bircher A.J., Bruze M., Cannavó A., Giménez-Arnau A., Gonçalo M., Goossens A. (2015). European Society of Contact Dermatitis guideline for diagnostic patch testing—Recommendations on best practice. Contact Dermat..

[152] Wilkinson M., Gonçalo M., Aerts O., Badulici S., Bennike N.H., Bruynzeel D., Dickel H., Garcia-Abujeta J.L., Giménez-Arnau A.M., Hamman C. (2019). The European baseline series and recommended additions: 2019. Contact Dermat..

[153] Davis M.D., Bhate K., Rohlinger A.L., Farmer S.A., Richardson D.M., Weaver A.L. (2008). Delayed patch test reading after 5 days: The Mayo Clinic experience. J. Am. Acad. Dermatol..

[154] Ahlgren C., Isaksson M., Möller H., Axéll T., Liedholm R., Bruze M. (2014). The necessity of a test reading after 1 week to detect late positive patch test reactions in patients with oral lichen lesions. Clin. Oral Investig..

[155] Van Amerongen C.C.A., Ofenloch R., Dittmar D., Schuttelaar M.L.A. (2019). New positive patch test reactions on day 7—The additional value of the day 7 patch test reading. Contact Dermat..

[156] Lindelöf B. (1992). Regional variations of patch test response in nickel-sensitive patients. Contact Dermat..

[157] Schittenhelm A., Stockinger W. (1925). Anaphylaxiestudien bei Mensch und Tier—IV. Mitteilung. Über die Idiosynkrasie gegen Nickel (“Nickelkrätze”) und ihre Beziehung zur Anaphylaxie. Z. Für Die Gesamte Exp. Med..

[158] Minné T., Calvin G. (1993). Concentration threshold of non-occluded nickel exposure in nickel-sensitive individuals and controls with and without surfactant. Contact Dermat..

[159] Muris J., Kleverlaan C.J., Feilzer A.J., Rustemeyer T. (2007). Sodium tetrachloropalladate (Na2[PdCl4]) as an improved test salt for palladium allergy patch testing. Contact Dermat..

[160] Muris J., Goossens A., Gonçalo M., Bircher A.J., Giménez-Arnau A., Foti C., Rustemeyer T., Feilzer A.J., Kleverlaan C.J. (2015). Sensitization to palladium and nickel in Europe and the relationship with oral disease and dental alloys. Contact Dermat..

[161] De Groot A.C. (2008). Patch testing. Test Conc. Veh..

[162] Gollhausen R., Przybilla B., Ring J. (1989). Reproducibility of patch tests. J. Am. Acad. Dermatol..

[163] Brasch J., Henseler T., Aberer W., Bäuerle G., Frosch P.J., Fuchs T., Fünfstück V., Kaiser G., Lischka G.G., Pilz B. (1994). Reproducibility of patch tests: A multicenter study of synchronous left- versus right-sided patch tests by the German Contact Dermatitis Research Group. J. Am. Acad. Dermatol..

[164] Bourke J.F., Batta K., Prais L., Abdullah A., Foulds I.S. (1999). The reproducibility of patch tests. Br. J. Dermatol..

[165] Ale S.I., Maibach H.I. (2004). Reproducibility of patch test results: A concurrent right-versus-left study using TRUE Testtm. Contact Dermat..

[166] Schaeffer A.C.V., Andersen K.E., Bindslev-Jensen C., Mortz C.G. (2016). The reproducibility of nickel, cobalt and chromate sensitization in patients tested at least twice in the period 1992-2014 with TRUE Test®. Contact Dermat..

[167] Dittmar D., Ofenloch R.F., Schuttelaar M.L.A. (2018). Persistence of contact allergy: A retrospective analysis. Contact Dermat..

[168] Hindsén M., Bruze M., Christensen O.B. (1999). Individual variation in nickel patch test reactivity. Am. J. Contact Dermat..

[169] Schalock P.C., Crawford G., Nedorost S., Scheinman P.L., Atwater A.R., Mowad C., Brod B., Ehrlich A., Watsky K.L., Sasseville D. (2016). Patch Testing for Evaluation of Hypersensitivity to Implanted Metal Devices: A Perspective From the American Contact Dermatitis Society. Dermatitis.

[170] Pacheco K.A. (2019). Allergy to Surgical Implants. Clin. Rev. Allergy Immunol..

[171] Hindsén M., Spiren A., Bruze M. (2005). Cross-reactivity between nickel and palladium demonstrated by systemic administration of nickel. Contact Dermat..

[172] Hammarlund E., Lewis M., Hansen S.G., I Strelow L., Nelson J.A., Sexton G.J., Hanifin J.M., Slifka M.K. (2003). Duration of antiviral immunity after smallpox vaccination. Nat. Med..

[173] Marraco S.A.F., Soneson C., Cagnon L., Gannon P.O., Allard M., Maillard S.A., Montandon N., Rufer N., Waldvogel S., Delorenzi M. (2015). Long-lasting stem cell–like memory CD8 + T cells with a naïve-like profile upon yellow fever vaccination. Sci. Transl. Med..

[174] Williams M., Bevan M.J. (2007). Effector and Memory CTL Differentiation. Annu. Rev. Immunol..

[175] Schoon J., Ort M.J., Huesker K., Geißler S., Rakow A. (2019). Diagnosis of Metal Hypersensitivity in Total Knee Arthroplasty: A Case Report. Front. Immunol..

[176] Saggau C., Scheffold A., Bacher P. (2021). Flow Cytometric Characterization of Human Antigen-Reactive T-Helper Cells. Methods Mol. Biol..

[177] Ständer S., Oppel E., Thomas P., Summer B. (2017). Evaluation of lymphocyte transformation tests as compared with patch tests in nickel allergy diagnosis. Contact Dermat..

[178] Von Blomberg-Van Der Flier M., Van Der Burg C.K.H., Pos O., Van De Plassche-Boers E.M., Bruynzeel D.P., Garotta G., Scheper R.J. (1987). In Vitro Studies in Nickel Allergy: Diagnostic Value of a Dual Parameter Analysis. J. Investig. Dermatol..

[179] Lisby S., Hansen L.H., Skov L., Menné T., Baadsgaard O. (1999). Nickel-induced activation of T cells in individuals with negative patch test to nickel sulphate. Arch. Dermatol. Res..

[180] Bechara R., Pollastro S., Azoury M.E., Szely N., Maillère B., de Vries N., Pallardy M. (2019). Identification and Characterization of Circulating Naïve CD4+ and CD8+ T Cells Recognizing Nickel. Front. Immunol..

[181] Blom L.H., Elrefaii S.A., Zachariae C., Thyssen J.P., Poulsen L.K., Johansen J.D. (2021). Memory T helper cells identify patients with nickel, cobalt, and chromium metal allergy. Contact Dermat..

[182] Moed H., von Blomberg M., Bruynzeel D.P., Scheper R., Gibbs S., Rustemeyer T. (2005). Improved detection of allergen-specific T-cell responses in allergic contact dermatitis through the addition of ’cytokine cocktails’. Exp. Dermatol..

[183] Al-Tawil N.G., Marcusson J.A., Möller E. (2008). HLA-class II restriction of the proliferative T lymphocyte responses to nickel, cobalt and chromium compounds. Tissue Antigens.

[184] Muris J., Kleverlaan C.J., Feilzer A.J., Valentine-Thon E. (2009). Reactivity to sodium tetrachloropalladate (Na2[PdCl4]) compared to PdCl2and NiCl2in lymphocyte proliferation tests. Allergy.

[185] Cristaudo A., Bordignon V., Petrucci F., Caimi S., De Rocco M., Picardo M., Fei P.C., Ensoli F. (2009). Release of Palladium from Biomechanical Prostheses in Body Fluids Can Induce or Support PD-Specific IFNγ T Cell Responses and the Clinical Setting of a Palladium Hypersensitivity. Int. J. Immunopathol. Pharmacol..

[186] Kimber I., Quirke S., Beck M. (1990). Attempts to identify the causative allergen in cases of allergic contact dermatitis using an in vitro lymphocyte transformation test. Toxicol. In Vitro.

[187] Cederbrant K., Anderson C., Andersson T., Marcusson-Ståhl M., Hultman P. (2003). Cytokine Production, Lymphocyte Proliferation and T-Cell Receptor Vβ Expression in Primary Peripheral Blood Mononuclear Cell Cultures from Nickel-Allergic Individuals. Int. Arch. Allergy Immunol..

[188] Moulon C., Vollmer J., Weltzien H.-U. (1995). Characterization of processing requirements and metal cross-reactivities in T cell clones from patients with allergic contact dermatitis to nickel. Eur. J. Immunol..

[189] Werfel T., Hentschel M., Kapp A., Renz H. (1997). Dichotomy of blood- and skin-derived IL-4-producing allergen-specific T cells and restricted V beta repertoire in nickel-mediated contact dermatitis. J. Immunol..

[190] Thomas P., Braathen L.R., Doerig M., Auboeck J., Nestle F., Werfel T., Willert H.G. (2009). Increased metal allergy in patients with failed metal-on-metal hip arthroplasty and peri-implant T-lymphocytic inflammation. Allergy.

[191] Vreeburg K.J., Groot K., Van Hoogstraten I.M., Von Blomberg M.E., Scheper R.J. (1991). Successful Induction of Allergic Contact Dermatitis to Mercury and Chromium in Mice. Int. Arch. Allergy Immunol..

[192] Mandervelt C., Clottens F., Demedts M., Nemery B. (1997). Assessment of the sensitization potential of five metal salts in the murine local lymph node assay. Toxicology.

[193] Basketter D.A., Lea L.J., Cooper K.J., A Ryan C., Gerberick G.F., Dearman R.J., Kimber I. (1999). Identification of metal allergens in the local lymph node assay. Arch. Phys. Med. Rehabil..

[194] Kligman A.M. (1989). The Identification of Contact Allergens by Human Assay III. The Maximization Test: A Procedure for Screening and Rating Contact Sensitizers. J. Investig. Dermatol..

[195] Basketter D. (2021). Nickel: Intrinsic Skin Sensitization Potency and Relation to Prevalence of Contact Allergy. Dermatitis.

[196] Schuttelaar M.L.A., Ofenloch R.F., Bruze M., Cazzaniga S., Elsner P., Gonçalo M., Naldi L., Svensson Å., Diepgen T.L. (2018). Prevalence of contact allergy to metals in the European general population with a focus on nickel and piercings: The EDEN Fragrance Study. Contact Dermat..

[197] Gibbs S., Kosten I., Veldhuizen R., Spiekstra S., Corsini E., Roggen E.L., Rustemeyer T., Feilzer A.J., Kleverlaan C.J. (2018). Assessment of metal sensitizer potency with the reconstructed human epidermis IL-18 assay. Toxicology.

[198] Matzinger P., Bevan M.J. (1977). Why do so many lymphocytes respond to major histocompatibility antigens?. Cell. Immunol..

[199] Jerne N.K. (1955). The natural-selection theory of antibody formation. Proc. Natl. Acad. Sci. USA.

[200] Burnet F.M. (1976). A Modification of Jerne’s Theory of Antibody Production using the Concept of Clonal Selection. CA Cancer J. Clin..

[201] Mason D. (1998). A very high level of crossreactivity is an essential feature of the T-cell receptor. Immunol. Today.

[202] Sewell A.K. (2012). Why must T cells be cross-reactive?. Nat. Rev. Immunol..

[203] Arstila T.P., Casrouge A., Baron V., Even J., Kanellopoulos J., Kourilsky P. (1999). A Direct Estimate of the Human αβ T Cell Receptor Diversity. Science.

[204] Robins H.S., Campregher P.V., Srivastava S.K., Wacher A., Turtle C.J., Kahsai O., Riddell S.R., Warren E., Carlson C.S. (2009). Comprehensive assessment of T-cell receptor β-chain diversity in αβ T cells. Blood.

[205] Wooldridge L., Ekeruche-Makinde J., Berg H.A.V.D., Skowera A., Miles J.J., Tan M.P., Dolton G., Clement M., Llewellyn-Lacey S., Price D.A. (2012). A Single Autoimmune T Cell Receptor Recognizes More Than a Million Different Peptides. J. Biol. Chem..

[206] Garboczi D.N., Ghosh P., Utz U., Fan Q.R., Biddison W.E., Wiley D.C. (1996). Structure of the complex between human T-cell receptor, viral peptide and HLA-A2. Nat. Cell Biol..

[207] Leem J., De Oliveira S.H.P., Krawczyk K., Deane C.M. (2018). STCRDab: The structural T-cell receptor database. Nucleic Acids Res..

[208] Clayton G.M., Wang Y., Crawford F., Novikov A., Wimberly B.T., Kieft J.S., Falta M.T., Bowerman N.A., Marrack P., Fontenot A.P. (2014). Structural Basis of Chronic Beryllium Disease: Linking Allergic Hypersensitivity and Autoimmunity. Cell.

[209] Yin L., Crawford F., Marrack P., Kappler J.W., Dai S. (2012). T-cell receptor (TCR) interaction with peptides that mimic nickel offers insight into nickel contact allergy. Proc. Natl. Acad. Sci. USA.

[210] Rudolph M.G., Stanfield R.L., Wilson I.A. (2006). How tcrs bind mhcs, peptides, and coreceptors. Annu. Rev. Immunol..

[211] Garcia K.C., Adams J.J., Feng D., Ely L.K. (2009). The molecular basis of TCR germline bias for MHC is surprisingly simple. Nat. Immunol..

[212] Feng D., Bond C.J., Ely L.K., A Maynard J., Garcia K.C. (2007). Structural evidence for a germline-encoded T cell receptor–major histocompatibility complex interaction ’codon’. Nat. Immunol..

[213] Birnbaum M., Mendoza J., Sethi D.K., Dong S., Glanville J., Dobbins J., Özkan E., Davis M.M., Wucherpfennig K.W., Garcia K.C. (2014). Deconstructing the Peptide-MHC Specificity of T Cell Recognition. Cell.

[214] Cole D., Bulek A.M., Dolton G., Schauenberg A.J., Szomolay B., Rittase W., Trimby A., Jothikumar P., Fuller A., Skowera A. (2016). Hotspot autoimmune T cell receptor binding underlies pathogen and insulin peptide cross-reactivity. J. Clin. Investig..

[215] Reiser J.-B., Grégoire C., Darnault C., Mosser T., Guimezanes A., Schmitt-Verhulst A.-M., Fontecilla-Camps J.C., Mazza G., Malissen B., Housset D. (2002). A T Cell Receptor CDR3β Loop Undergoes Conformational Changes of Unprecedented Magnitude Upon Binding to a Peptide/MHC Class I Complex. Immunity.

[216] Siewert K., Malotka J., Kawakami N., Wekerle H., Hohlfeld R., Dornmair K. (2012). Unbiased identification of target antigens of CD8+ T cells with combinatorial libraries coding for short peptides. Nat. Med..

[217] Arakawa A., Siewert K., Stöhr J., Besgen P., Kim S.-M., Rühl G., Nickel J., Vollmer S., Thomas P., Krebs S. (2015). Melanocyte antigen triggers autoimmunity in human psoriasis. J. Exp. Med..

[218] Mazza C., Auphan-Anezin N., Grégoire C., Guimezanes A., Kellenberger C., Roussel A., Kearney A., Van Der Merwe P.A., Schmitt-Verhulst A.-M., Malissen B. (2007). How much can a T-cell antigen receptor adapt to structurally distinct antigenic peptides?. EMBO J..

[219] Garcia K.C., Degano M., Pease L.R., Huang M., Peterson P.A., Teyton L., Wilson I.A. (1998). Structural Basis of Plasticity in T Cell Receptor Recognition of a Self Peptide-MHC Antigen. Science.

[220] Baker B.M., Scott D.R., Blevins S.J., Hawse W.F. (2012). Structural and dynamic control of T-cell receptor specificity, cross-reactivity, and binding mechanism. Immunol. Rev..

[221] Ding Y.-H., Baker B.M., Garboczi D.N., E Biddison W., Wiley D.C. (1999). Four A6-TCR/Peptide/HLA-A2 Structures that Generate Very Different T Cell Signals Are Nearly Identical. Immunity.

[222] Armstrong K.M., Piepenbrink K.H., Baker B.M. (2008). Conformational changes and flexibility in T-cell receptor recognition of peptide–MHC complexes. Biochem. J..

[223] Kjer-Nielsen L., Clements C.S., Purcell A., Brooks A., Whisstock J., Burrows S., McCluskey J., Rossjohn J. (2003). A Structural Basis for the Selection of Dominant αβ T Cell Receptors in Antiviral Immunity. Immunity.

[224] Borbulevych O.Y., Piepenbrink K.H., Gloor B.E., Scott D.R., Sommese R.F., Cole D.K., Sewell A.K., Baker B.M. (2009). T Cell Receptor Cross-reactivity Directed by Antigen-Dependent Tuning of Peptide-MHC Molecular Flexibility. Immunity.

[225] Lang H., Jacobsen H., Ikemizu S., Andersson C., Harlos K., Madsen L., Hjorth P., Sondergaard L., Svejgaard A., Wucherpfennig K. (2002). A functional and structural basis for TCR cross-reactivity in multiple sclerosis. Nat. Immunol..

[226] Macdonald W.A., Chen Z., Gras S., Archbold J., Tynan F.E., Clements C.S., Bharadwaj M., Kjer-Nielsen L., Saunders P.M., Wilce M.C. (2009). T Cell Allorecognition via Molecular Mimicry. Immunity.

[227] Harkiolaki M., Holmes S.L., Svendsen P., Gregersen J.W., Jensen L.T., McMahon R., Friese M.A., van Boxel G., Etzensperger R., Tzartos J.S. (2009). T Cell-Mediated Autoimmune Disease due to Low-Affinity Crossreactivity to Common Microbial Peptides. Immunity.

[228] Nelde A., Bilich T., Heitmann J.S., Maringer Y., Salih H.R., Roerden M., Lübke M., Bauer J., Rieth J., Wacker M. (2021). SARS-CoV-2-derived peptides define heterologous and COVID-19-induced T cell recognition. Nat. Immunol..

[229] Bacher P., Rosati E., Esser D., Martini G.R., Saggau C., Schiminsky E., Dargvainiene J., Schröder I., Wieters I., Khodamoradi Y. (2020). Low-Avidity CD4+ T Cell Responses to SARS-CoV-2 in Unexposed Individuals and Humans with Severe COVID-19. Immunity.

[230] Pichler W.J. (2002). Pharmacological interaction of drugs with antigen-specific immune receptors: The p-i concept. Curr. Opin. Allergy Clin. Immunol..

[231] Moulon C., Wild D., Weltzien H.U., Dormoy A. (1998). MHC-Dependent and -Independent Activation of Human Nickel-Specific CD8+ Cytotoxic T Cells from Allergic Donors11This work was presented in part at the 26th Annual Meeting of the European Society of Dermatologic Research, Amsterdam, September 19–22, 1996. J. Investig. Dermatol..

[232] Thierse H.-J., Moulon C., Allespach Y., Zimmermann B., Doetze A., Kuppig S., Wild D., Herberg F., Weltzien H.U. (2004). Metal-Protein Complex-Mediated Transport and Delivery of Ni2+ to TCR/MHC Contact Sites in Nickel-Specific Human T Cell Activation. J. Immunol..

[233] Falta M.T., Pinilla C., Mack D.G., Tinega A.N., Crawford F., Giulianotti M., Santos R., Clayton G.M., Wang Y., Zhang X. (2013). Identification of beryllium-dependent peptides recognized by CD4+ T cells in chronic beryllium disease. J. Exp. Med..

[234] Romagnoli P., Labhardt A., Sinigaglia F. (1991). Selective interaction of Ni with an MHC-bound peptide. EMBO J..

[235] Romagnoli P., A Spinas G., Sinigaglia F. (1992). Gold-specific T cells in rheumatoid arthritis patients treated with gold. J. Clin. Investig..

[236] De Wall S.L., Painter C., Stone J.D., Bandaranayake R., Wiley D.C., Mitchison T.J., Stern L.J., Dedecker B.S. (2006). Noble metals strip peptides from class II MHC proteins. Nat. Chem. Biol..

[237] Nasorri F., Sebastiani S., Mariani V., Girolomoni G., Cavani A., De Pità O., Puddu P. (2002). Activation of Nickel-Specific CD4+ T Lymphocytes in the Absence of Professional Antigen-Presenting Cells. J. Investig. Dermatol..

[238] Shigematsu H., Kumagai K., Suzuki M., Eguchi T., Matsubara R., Nakasone Y., Nasu K., Yoshizawa T., Ichikawa H., Mori T. (2020). Cross-Reactivity of Palladium in a Murine Model of Metal-induced Allergic Contact Dermatitis. Int. J. Mol. Sci..

[239] Griem P., Panthel K., Kalbacher H., Gleichmann E. (1996). Alteration of a model antigen by Au(III) leads to T cell sensitization to cryptic peptides. Eur. J. Immunol..

[240] Griem P., von Vultée C., Panthel K., Best S.L., Sadler P.J., Shaw III C.F. (1998). T cell cross-reactivity to heavy metals: Identical cryptic peptides may be presented from protein exposed to different metals. Eur. J. Immunol..

[241] Moulon C., Choleva Y., Thierse H.-J., Wild D., Weltzien H.U. (2003). T Cell Receptor Transfection Shows Non-HLA-Restricted Recognition of Nickel by CD8+ Human T Cells to be Mediated by αβ T Cell Receptors. J. Investig. Dermatol..

[242] Comstedt L.R., Dahlin J., Bruze M., Åkesson A., Hindsén M., Pontén A., Isaksson M., Svedman C. (2020). Prevalence of contact allergy to metals: Nickel, palladium, and cobalt in Southern Sweden from 1995–2016. Contact Dermat..

[243] Gawkrodger D.J., Lewis F.M., Shah M. (2000). Contact sensitivity to nickel and other metals in jewelry reactors. J. Am. Acad. Dermatol..

[244] Lagrelius M., Wahlgren C.-F., Matura M., Kull I., Lidén C. (2015). High prevalence of contact allergy in adolescence: Results from the population-based BAMSE birth cohort. Contact Dermat..

[245] Lisi P., Brunelli L., Stingeni L. (2003). Co-sensitivity between cobalt and other transition metals. Contact Dermat..

[246] Kanerva L., Kerosuo H., Kullaa A., Kerosuo E. (1996). Allergic patch test reactions to palladium chloride in schoolchildren. Contact Dermat..

[247] Santucci B., Cannistraci C., Cristaudo A., Picardo M. (1996). Multiple sensitivities to transition metals: The nickel palladium reactions. Contact Dermat..

[248] Kinbara M., Nagai Y., Takano-Yamamoto T., Sugawara S., Endo Y. (2011). Cross-reactivity among some metals in a murine metal allergy model. Br. J. Dermatol..

[249] Santucci B., Cristaudo A., Cannistraci C., Picardo M. (1995). Interaction of palladium ions with the skin. Exp. Dermatol..

[250] Pistoor F.H.M., Kapsenberg M.L., Bos J.D., Meinardi M.M.H.M., E Von Blomberg M., Scheper R.J., Mary B. (1995). Cross-Reactivity of Human Nickel-Reactive T-Lymphocyte Clones with Copper and Palladium. J. Investig. Dermatol..

[251] Löfström A., Wigzell H. (1986). Antigen specific human T cell lines specific for cobalt chloride. Acta Derm. Venereol..

[252] Thomssen H., Hoffmann B., Schank M., Höhler T., Thabe H., Büschenfelde K.H.M.Z., Märker-Hermann E. (2001). Cobalt-specific T lymphocytes in synovial tissue after an allergic reaction to a cobalt alloy joint prosthesis. J. Rheumatol..

